# Showcasing MESMER‐X: Spatially Resolved Emulation of Annual Maximum Temperatures of Earth System Models

**DOI:** 10.1029/2022GL099012

**Published:** 2022-08-30

**Authors:** Y. Quilcaille, L. Gudmundsson, L. Beusch, M. Hauser, S. I. Seneviratne

**Affiliations:** ^1^ Institute for Atmospheric and Climate Science Department of Environmental Systems Science ETH Zurich Zurich Switzerland; ^2^ Now at: Federal Office of Meteorology and Climatology MeteoSwiss Zurich Switzerland

**Keywords:** climate extremes, emulation, modeling, scenarios, earth system, climate change

## Abstract

Emulators of Earth System Models (ESMs) are complementary to ESMs by providing climate information at lower computational costs. Thus far, the emulation of spatially resolved climate extremes has only received limited attention, even though extreme events are one of the most impactful aspects of climate change. Here, we propose a method for the emulation of local annual maximum temperatures, with a focus on reproducing essential statistical properties such as correlations in space and time. We test different emulator configurations and find that driving the emulations with global mean surface temperature offers an optimal compromise between model complexity and performance. We show that the emulations can mimic the temporal evolution and spatial patterns of the underlying climate model simulations and are able to reproduce their natural variability. The general design and the good performance for annual maximum temperatures suggest that the proposed methodology can be applied to other climate extremes.

## Key Points

- We present a method for the emulation of spatially resolved annual maximum temperatures from Earth System Models
- The emulator reproduces statistical properties and correlations in space and time and can be extended to other extreme indicators
- The method exhibits good performance for annual maximum temperatures

## 1 Introduction

The impacts of climate change impact the entire social and economic system (IPCC, 2014, 2021). In particular, changes in climate extremes count among the most impactful consequences of climate change. Climate extremes are substantially affected by human‐induced climate change (Seneviratne et al., 2021). For example, the annual average losses to weather‐related disasters in 2020 USD were 168 billion per year over 2001–2010 and have increased to 248 billion per year over 2011–2020 (AON, 2020). Climate extremes affect numerous economic sectors, for instance, the agriculture (Sivakumar et al., 2005; Vogel et al., 2019) or the energy sector (Perera et al., 2020; Schaeffer et al., 2012). Not only do climate extremes have direct consequences on food or energy security (Hasegawa et al., 2021), but they can also have indirect impacts on societies due to feedbacks with societal drivers (Raymond et al., 2020). Even if climate change were limited to 1.5°C, changes in climate extremes would remain a crucial issue (Seneviratne et al., 2018), and society will be impacted in many aspects (IPCC, 2018).

Earth System Models (ESMs) are used to derive climate change projections and the associated climate extremes (Collins et al., 2013; Flato et al., 2013; Lee et al., 2021). These outputs are crucial to assess what consequences changes in climate extremes would have on society (Rosenzweig et al., 2017). However, simulating climate change with ESMs is computationally expensive, hindering their use in exploring new scenarios while characterizing internal climate variability.

ESM emulators have been developed for a quicker assessment of climate change in response to given scenario pathways. A large class of emulators, termed “simple climate models” or “reduced complexity models” provide projections of key variables of the Earth system such as global mean temperature (Nicholls et al., 2020, 2021); however, they do not provide local information, which is essential for studying climate impacts. A second class of emulators derives spatially resolved climate responses (spatially resolved emulators), such as the recently developed Modular Earth System Model Emulator with spatially Resolved output (MESMER) (Beusch et al., 2020). Spatially resolved emulators usually rely on some version of pattern scaling to derive local responses from global variables (Alexeeff et al., 2018; Fordham et al., 2012; Herger et al., 2015; Lynch et al., 2017; Mitchell, 2003). While other approaches exist (Castruccio et al., 2014; Holden et al., 2014), pattern scaling shows good performances despite its simplicity (Tebaldi & Arblaster, 2014; Tebaldi & Knutti, 2018). For the representation of natural variability in spatially resolved emulators, there is no single most established method. Some emulators resample actual ESM fields (Alexeeff et al., 2018; McKinnon et al., 2017), some resample principle components with perturbed phases (Link et al., 2019), and others rely on autoregressive processes with spatially correlated innovations (Beusch et al., 2020; Nath et al., 2021). Almost all spatially resolved emulation approaches have been developed to emulate mean quantities. However, to assess the impacts of climate change for diverse emission pathways, emulations of climate extremes are also needed. A first step in this direction has been made by Tebaldi et al. (2020), who use pattern scaling to emulate the average evolution of climate extremes, but do not consider natural variability. Thus, an emulator of distributions of climate extremes is still lacking. In this paper, we propose a new method for spatially resolved emulation of climate extremes that accounts for both the spatio‐temporal structure and their internal variability. Building on the MESMER emulator (Beusch et al., 2020), the presented approach is referred to as MESMER‐X.

## 2 Data

Simulations from 18 ESMs contributing to the Scenario Model Intercomparison Project (ScenarioMIP; O'Neill et al., 2016; Eyring et al., 2016) of CMIP6 are considered (Table S1 in Supporting Information S1). In particular, we use one single ensemble member of ESMs which provides data for concentration‐driven historical (Meinshausen et al., 2017) and for at least two of the five scenarios SSP1‐1.9, SSP1‐2.6, SSP2‐4.5, SSP3‐7.0, and SSP5‐8.5 (Meinshausen et al., 2020). Moreover, we retain only ESMs providing daily maximum near‐surface air temperature, near‐surface air temperature, and downward surface sensible heat flux over the ocean.

All simulations are interpolated to the same 2.5° × 2.5° grid using second‐order conservative remapping for the two temperatures and inverse distance‐weighted average for the heat flux (Brunner, Hauser, et al., 2020). Spatially resolved local annual maximum temperature (TXx) is calculated as the annual maximum of the daily maximum temperature. The anomaly of the local annual maximum temperature is defined by subtracting its 1850–1900 local mean. The global mean surface air temperature (GSAT) and the global downward heat flux in sea water (GHFDS) are derived by first averaging spatially the annual mean, then their anomalies are also calculated by subtracting their 1850–1900 mean.

In Section 4, both the global trend and global variability of GSAT and GHFDS are used to identify adequate drivers for the emulations. These two components are decomposed into a forced and a variability component using a locally weighted scatterplot smoothing (LOWESS), which additionally accounts for volcanic eruptions in GSAT as explained in Beusch et al. (2020).

Some results are aggregated to sub‐continental regions defined for the sixth Assessment Report of IPCC regions (Iturbide et al., 2020).

## 3 A Method for the Emulation of Climate Extremes

### 3.1 Statistical Distribution of Local Climate Extremes

Climate variables can be characterized by stochastic and dynamic processes, and climate extremes are rare values or events of these climate variables, in the tail of their probability distribution (Storch & Zwiers, 1999; Wilks, 2011). This definition implies that changes in the distribution of climate variables will also affect the distribution of climate extremes. For instance, if the local annual mean surface temperature increases, it is likely that the local annual maximum surface temperature will increase as well. For example, regional anomalies of annual maximum temperature have been found to scale linearly with anomalies in GSAT (Seneviratne et al., 2016, 2018; Wartenburger et al., 2017). At a local scale, this scaling still performs well, although internal variability has to be addressed (Tebaldi et al., 2020). Here, we exploit this dependency by modeling the distribution of annual maximum temperatures at each grid‐cell conditional on GSAT and other globally available predictor variables.

We write $\Delta X_{s,t}$ the local anomaly of TXx at each point in space s and timestep t. We assume here that $\Delta X_{s,t}$ follows a Generalized Extreme Value (GEV) distribution, because TXx is a block maxima (Coles, 2001; Wilks, 2011) and we note that the GEV has been successfully used to model TXx elsewhere (Hauser et al., 2016; Huang et al., 2016; Kim et al., 2020). We further assume that the location, scale, and shape parameters of the GEV are point‐dependent and timestep‐dependent, written as $\mu_{s,t}$, $\sigma_{s,t}$, and $\xi_{s,t}$. More precisely, we disentangle these dependencies by assuming that these parameters follow the point‐dependent functions $f_{s}$, $g_{s}$, and $h_{s}$, taking time‐dependent covariates $\Delta V_{t}$ as input. These covariates are defined as time series of anomalies in global climate variables such as GSAT, but other globally available covariates can be considered. We define the emulator configuration E as the set of Equation 1. Examples are shown in Section 4.1.

(1) $$E:\left\{\begin{array}{l} \Delta X_{s,t}\;∼\;GEV\left(\mu_{s,t},\;\sigma_{s,t},\xi_{s,t}\right)\\ \mu_{s,t}=f_{s}\left(\Delta V_{t}\right)\\ \sigma_{s,t}=g_{s}\left(\Delta V_{t}\right)\\ \xi_{s,t}=h_{s}\left(\Delta V_{t}\right) \end{array}\right.\;$$

For each ESM, the coefficients in the functions $f_{s}$, $g_{s}$, and $h_{s}$ are estimated by minimizing the negative log‐likelihood over the considered scenarios and ensemble members. To ensure the convergence of the fit, the local first guess of the coefficients for the parameters is optimized using an adapted method of moments as described in Text S1 of Supporting Information S1.

### 3.2 Spatio‐Temporal Coherent Sampling of Climate Extremes

For approximating internal climate variability, we aim at devising a stochastic model that produces spatially and temporally correlated samples of TXx that follow Equation 1. To this end, we follow (Beusch et al., 2020) that parameterizes internal climate variability in annual mean temperature anomalies using a local auto‐regressive processes with spatially correlated innovations (Humphrey & Gudmundsson, 2019). A key assumption of this approach is that the variability is stationary in time and approximately normally distributed. This is however not the case for residuals of the model in Equation 1. Instead, we employ the fitted distributions to transform TXx to a standard normal distribution using the probability integral transform (Angus, 1994; Gneiting et al., 2007; Gudmundsson et al., 2012). For the emulator configuration defined in Section 3.1, the GEV of TXx and its cumulative distribution function ${ℱ}_{GEV}\left(\Delta X_{s,t}|\Delta V_{t},f_{s},g_{s},h_{s}\right)$ are known over the full data set. We define ${ℱ}_{N}^{-1}$ as the quantile function of the standard normal distribution. Using these two functions, we transform $\Delta X_{s,t}$ to a standard normally distributed transformed TXx, that we write as $\Phi_{s,t}$.

(2) $$\Phi_{s,t}=\;{ℱ}_{N}^{-1}\left({ℱ}_{GEV}\left(\Delta X_{s,t}|\Delta V_{t},f_{s},g_{s},h_{s}\right)\right)\;$$

While $\Delta X_{s,t}$ follows a non‐stationary GEV distribution, $\Phi_{s,t}$ has a normal distribution stationary in time, thus respecting the required conditions (Beusch et al., 2020; Humphrey & Gudmundsson, 2019). Note that no information is lost in this transformation, because the GEV associated with $\Phi_{s,t}$ is known at each point s and timestep t, which will be used in Section 3.3. We train on $\Phi_{s,t}$ a local auto‐regressive process of order 1 with parameters $\gamma_{s,0}$ and $\gamma_{s,1}$, with spatially correlated innovations following Equation 3.

(3) $$\Phi_{s,t}=\;\gamma_{s,0}+\;\gamma_{s,1}\Phi_{s,t-1}+\upsilon_{s,t}\;$$

The spatial innovations $\upsilon_{s,t}$ are sampled from a multivariate normal distribution deduced from an empirically estimated and localized covariance matrix that represents spatial dependence between points as explained in Beusch et al. (2020).

### 3.3 Emulating Spatio‐Temporally Correlated Climate Extremes

Emulations for any scenario can be created if time series of anomalies of global climate variables $\Delta V_{t}$, such as GSAT or GHFDS are provided for it. Together with Equation 1, it defines the distribution of TXx at any point in time.

Using the auto‐regressive processes with spatially correlated innovations, we draw realizations $\Phi_{s,t,e}$ for all points s, timesteps t, and index of emulation e. These realizations represent TXx with its natural variability transformed onto a standard normal distribution, and independent from the scenario. We then backtransform $\Phi_{s,t,e}$ onto the distribution of TXx using its quantile function of the GEV, ${ℱ}_{GEV}^{-1}$, and the cumulative distribution function of the standard normal distribution, ${ℱ}_{N}$, leading to the emulations of TXx written $\Delta X_{s,t,e}$:

(4) $$\Delta X_{s,t,e}=\;{ℱ}_{GEV}^{-1}\left({ℱ}_{N}\left(\Phi_{s,t,e}\right)|\Delta V_{t},f_{s},g_{s},h_{s}\;\right)\;$$

## 4 Emulating Extreme Temperatures Under Climate Change

### 4.1 Selecting and Evaluating Emulator Configurations

**Figure 1:**
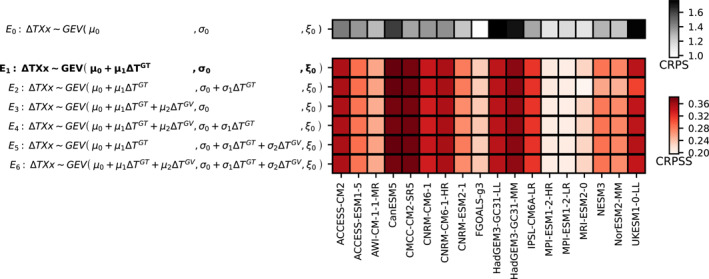
Selection of an emulator configuration. The first row shows the CRPS for the emulator configuration *E*_0_ used as a reference. A high CRPS indicates that there is a stronger climate signal in the distribution of TXx. On the following rows, the CRPSS with reference to the emulator configuration *E*_0_ is shown for each emulator configuration and ESM. A high CRPSS indicates that there is a better reproduction of the climate signal.

For each of the 18 ESMs, we consider historical simulations over 1850–2014 and all available scenarios over 2015–2100 to calibrate the emulator using different sets of explanatory variables ($\Delta V_{t}$) that can linearly affect the parameters of the underlying distribution. Figure 1 lists a selection of setups, where the first row corresponds to a baseline model with no covariates. This baseline model is used for benchmarking the others. The two globally available explanatory variables considered are the global trend $\Delta T^{GT}$ and the global variability $\Delta T^{GV}$ of the GSAT anomaly (Section 2). The global trend $\Delta T^{GT}$ is meant to capture the signal from global warming, while the global variability $\Delta T^{GV}$ represents internal variability that is relevant at the global scale.

For evaluating the skill of the emulator an ensemble of 1,000 realizations is drawn for each of the considered setups. Subsequently, the ability of the emulator to reproduce the ESM's TXx anomaly distribution is evaluated using the Continuous Rank Probability Score (CRPS) and the CRPS Skill Score (CRPSS), commonly used in atmospheric sciences (Jolliffe & Stephenson,  2012; Wilks, 2011). The CRPS measures the quadratic discrepancy between the cumulative distribution function of the emulations to the one of the ESM and is computed for each point of the sample (Equation 5). The skill score is defined relatively to the benchmark $E_{0}$ (Equation 6), that is to say an emulator that does not account for climate change.

(5) $$CRPS^{E}{\left(\Delta X_{s,t,e}^{E},\Delta X_{s,t}\right)}_{s,t}=\overset{+\infty }{\underset{-\infty }{\int }}\frac{1}{N_{e}}\overset{N_{e}}{\underset{e=1}{\sum }}{\left[1\left(\Delta X\geq \Delta X_{s,t,e}^{E}\right)-1\left(\Delta X\geq \Delta X_{s,t}\right)\right]}^{2}d\Delta X\;$$

(6) CRPSSs,tE=1−CRPSs,tECRPSs,tE0

Figure 1 shows the global average CRPS for the baseline model ($E_{0},\;$ first row) and the CRPSS for all other configurations (remaining rows). For the configuration $E_{1}$, only the location parameter depends on $\Delta T^{GT}$. Compared to $E_{0}$, it reduces the CRPS on average by about 28%. The ESMs with a low CRPS in $E_{0}$ (e.g., FGOALS‐g3) have their TXx less influenced by climate change than those with a higher CRPS such as HadGEM3‐GC31‐LL, HadGEM3‐GC31‐MM, and UKESM1‐0‐LL. Those ESMs with a low CRPS have a low CRPSS as well because the new emulator configuration brings comparably little improvement. However, those with a higher CRPS benefit from a stronger reduction in their CRPS by including a dependency of the GEV to climate change. On the following rows, further combinations are investigated. However, these more complex models have only marginal gains, or even lead to a reduction in the capacity of the emulator to reproduce the climate signal (e.g., $E_{2}$). These results are confirmed by comparing the global distribution of CRPS using Mann–Whitney *U*‐tests: adding additional terms for the emulation of TXx either brings no significant improvement, or slightly reduces the quality of the emulations. We further observe that the emulator configurations $E_{2}$ to $E_{6}$ bring improvement only in some regions of the Earth (not shown), while they hamper the fit in many others, which is consistent with (Kharin & Zwiers, 2005; Kim et al., 2020).

In Figure 1, results for historical and scenario simulations are aggregated for simplicity, but details over each scenario are provided in Figures S1–S7 in Supporting Information S1. Figures S1–S7 in Supporting Information S1 feature as well additional emulator configurations and make use of the GHFDS. These figures present additional emulator configurations that are described in Text S2 in Supporting Information S1. For clarity, the global mean assessment is shown in Figure 1, but grid‐cell level CRPSS is additionally depicted in Figures S8–S25 in Supporting Information S1, that allows to assess regional differences in model skill.

In summary, this analysis shows that the emulator configuration $E_{1}$ provides the best compromise of simplicity and quality for emulations of TXx. The results in the rest of the paper will therefore use $E_{1}$, that is, with only the location parameter of the GEV varying linearly with $\Delta T^{GT}$.

### 4.2 Example of Emulations

**Figure 2:**
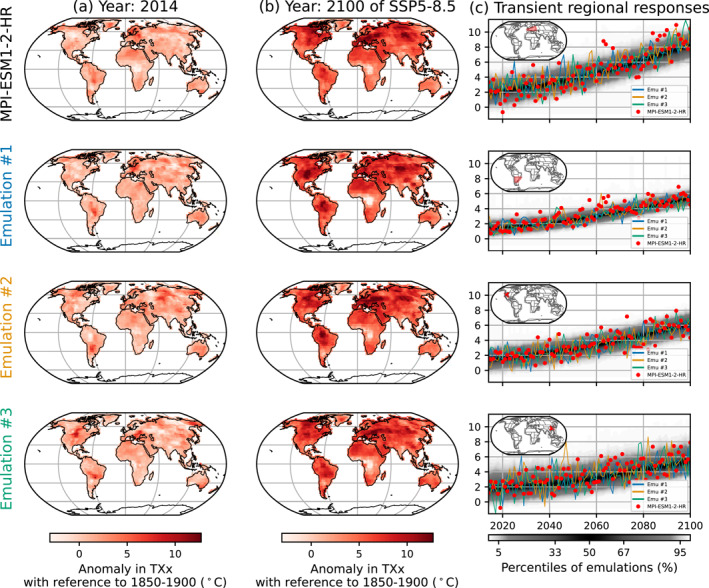
Example of emulations. The local anomalies in TXx are shown for MPI‐ESM1‐2‐HR and three emulations for the years 2014 and 2100, in columns (a) and (b), respectively. The transient regional response from 2014 to 2100 is shown in column (c) for selected regions and points. The first and second rows of column (c) are the regions West and Central Europe and South‐East of South America. The third and fourth rows of column (c) are two points located in the United States and China. Column (c) shows MPI‐ESM1‐2‐HR, the same three emulations shown in maps, and the density of the 1,000 emulations.

Figure 2 shows an example of our results for MPI‐ESM1‐2‐HR. We compare the maps of the anomaly in TXx of the ESM (topmost row) with three of the 1,000 emulations for this ESM. We show the years 2014 and 2100 to compare ESM values with emulations under current and high warming conditions.

The emulations capture the general spatial features in TXx well, be it in 2014 or in 2100, but no exact match to the ESM simulation which is expected since they sample internal variability. For example, both the emulations and the ESM simulate the positive anomaly over Eastern Europe and the center of South America or the lower anomaly over Central Africa. Because each emulation includes natural variability, some features are more pronounced than others, such as the high anomaly in the center of North America.

To further investigate temporal dynamics, we represent the transient response in two specific regions and two specific points as detailed in Figure 2. Overall, the emulations are in good agreement with the ESM. The ensemble of emulations correctly encompasses the realization by MPI‐ESM1‐2‐HR. Figures S26–S43 in Supporting Information S1 show the same results for the other considered ESMs.

### 4.3 Evaluation of Regional Performance

To quantify the performance of the emulator on a regional level, we follow (Beusch et al., 2020) and compare regional percentiles of the emulations to the ESMs. For each ESM and emulation, the anomalies in TXx are averaged over the AR6 regions. Next, we calculate the 95%, 50%, and 5% percentiles of the regional emulations and count how often the regional values of the ESM exceed these thresholds over each available scenario. By comparing the deviation in these regional percentiles, we diagnose the reproduction of the regional statistical distribution of the ESM.

**Figure 3:**
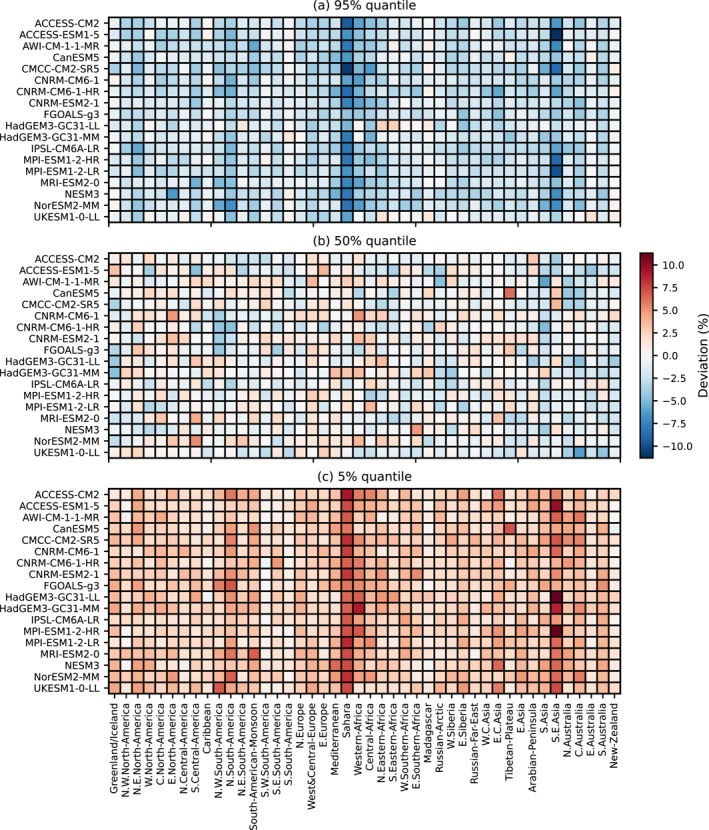
Regional deviations of ESMs from the 5% (a), 50% (b), and 95% (c) quantiles of the emulations. Red (blue) indicates that the quantile of the emulations is higher (lower) than the one of ESM, because the ESM is more frequently below (above) the quantile than expected.

Figure 3 shows the regional deviation in the quantiles. Panel (a) shows that the emulations cross the 95% percentile of the ESM too rarely, while panel (c) shows the opposite for the 5% percentile. This means that the emulation is underdispersive, which is expected because the method used to sample spatially correlated innovations dampens spatial correlations as a function of distance between grid points, as described in Beusch et al. (2020). The deviation of the quantiles is stronger in South‐East Asia and in the Sahara, suggesting that some local processes or teleconnections are not accounted for in this emulator. Yet, overall the performance remains good: the regional deviations are below 5% in most of the cases. For the quantiles 95%, 50%, and 5%, they are below 5% in respectively 93%, 99%, and 92% of the ESM‐region combinations. The average of the regional deviations across regions and ESMs is −2.4%, −0.3%, and 2.9%.

### 4.4 Example Application

Some of the considered ESMs only provide a subset of the scenarios SSP1‐1.9, SSP1‐2.6, SSP2‐4.5, SSP3‐7.0, and SSP5‐8.5, which hinders the evaluation of a distribution of anomalies in TXx based on all ESMs. Here we employ the newly developed emulator to fill in this gap, by transferring the parameters that were trained on the available data to trajectories of globally available explanatory variables ($\Delta V_{t}$) of the missing scenarios.

**Figure 4:**
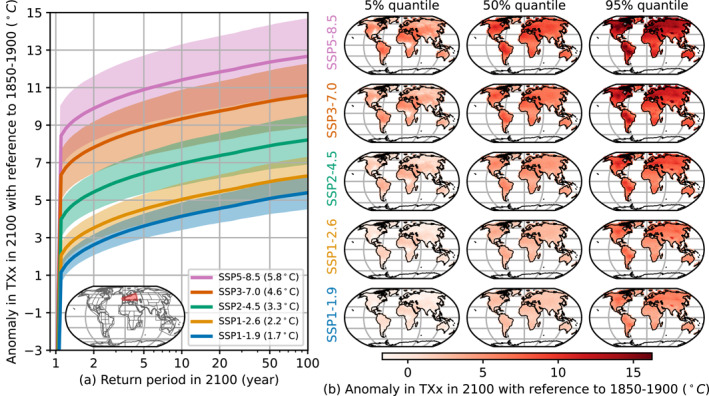
Illustration of the ensemble formed by 1,000 emulations of the 18 trained ESMs, applied over common scenarios. The return periods in 2100 in West and Central Europe of each ESM are shown in (a) through their mean and one standard deviation range. In the legend, the anomaly in GSAT in 2100 of each scenario is provided. (b) Shows the local 5%, 50%, and 95% quantiles in 2100, all ESMs being pooled together. Each row corresponds to a scenario.

In the selected configuration, MESMER‐X can emulate scenarios if time series of $\Delta T^{GT}$ are provided. For each of the scenarios, we average $\Delta T^{GT}$ over all ESMs that have run the scenario. These averaged $\Delta T^{GT}$ are used as common drivers to create emulations for all ESMs for every scenario. For each of the 18 ESMs, we calculate an ensemble of 1,000 realizations that combines two sources of dispersion: the local variability in TXx modeled by the ESM and the uncertainty in this modeling by ESMs, also termed “regional climate sensitivity” (Seneviratne & Hauser, 2020). Yet, it does not encompass the global uncertainty due to the different global climate sensitivities of the ESMs. Additionally, we are not weighting ESMs according to their performances nor accounting for ESM‐interdependencies (Abramowitz et al., 2019; Brunner, Pendergrass, et al., 2020). We calculate the return periods under each emulated ESM and scenario. Figure 4a shows their mean and standard deviation over the emulated ESMs, thus representing the uncertainty induced by the different ESMs' different representation of natural variability. In Figure 4b, the distributions pool all emulations together, merging natural variability and its uncertainty.

Figure 4a shows that in West and Central Europe, a TXx anomaly of 5°C would happen about once in 40 years in 2100 under SSP1‐1.9, but every 10 years under SSP1‐2.6 and every one or 2 years under SSP2‐4.5. This result is consistent with how climate extremes are projected for 1.5°C (Seneviratne et al., 2018) and the change from 1.5°C to 2°C (Hoegh‐Guldberg et al., 2018).

Quantiles of spatially resolved emulations for 2100 are shown in Figure 4b. The 95% quantile of SSP1‐1.9 seems overall only slightly higher than the 5% quantile of SSP5‐8.5. Broadly speaking, it would suggest that anomalies in TXx that had only a 5% chance to occur or be exceeded in SSP1‐1.9 in 2100, would have their probability increase to 95% in SSP5‐8.5.

## 5 Discussion and Conclusions

This study has introduced a method for the emulation of climate extremes under climate change, used to extend the MESMER emulator (Beusch et al., 2020) to MESMER‐X. This method does not only reproduce the mean evolution of climate extremes but also their distribution. Besides, it accounts for their spatial and temporal features.

Fits of non‐stationary GEV for TXx have already been performed using different covariates on the location before (Hauser et al., 2016; Wehner, 2020; Wehner et al., 2020; Zwiers et al., 2011). Here, we leverage this approach to model the distribution of TXx at each point conditional on global covariates. The proposed method is improved by its greater versatility in the use of covariates and in its sampling of stochastic realizations of time‐series fields. We show that the emulator mimics local annual maximum ESM temperatures well, with an underdispersion below 5% for most regions and ESMs.

In this study, a single ensemble member is used for each ESM, because it is sufficient to train the key properties of the models (Beusch et al., 2020) and because our main focus is to introduce a novel framework for emulating climate extremes. Nevertheless, we note that out‐of‐sample validation can increase the confidence in the resulting emulations. To this end, future studies could make use of more ensemble members, to evaluate the performance of MESMER‐X on out‐of‐samples runs. Furthermore, additional ensemble members, if available, are also expected to contribute to a statistically more robust estimation of the parameters of MESMER‐X.

This method is designed to be directly applied to other indicators of climate extremes, as long as their distribution can be parametrized by a GEV. It concerns any extrema of a climate variable over a year, for instance, annual minimum temperature, annual maximum precipitation, or annual maxima of the Fire Weather Index. Moreover, the framework can be adapted to different distributions appropriate for other indicators, such as a Poisson distribution for counting extreme events (Wilks, 2011) or a generalized Pareto distribution for climate extremes based on peak‐over‐threshold exceedances (Coles, 2001; Naveau et al., 2005). The parameters of these distributions may vary with any combination of global drivers to improve the quality of the emulator configuration.

Similar to MESMER (Beusch, Nauels, et al., 2022; Beusch, Nicholls, et al., 2022), MESMER‐X could be coupled to a simple climate model in future work to gain the ability to transform any emission scenario into local annual climate extremes in a fast and probabilistic way. Such an emulator chain could be used to provide detailed climate information into integrated assessment models, for instance, to assess how climate extremes affect different transformation pathways.

6

## Supporting information

Supporting Information S1

## Data Availability

Data from CMIP6 are available at https://esgf-node.llnl.gov/search/cmip6/ (last access: July 30, 2022). Detailed data for the search query are as follows: Experiment ID (historical, ssp119, ssp1226, ssp245, ssp370, and ssp585) and variable (tas, tasmax, and hfds). Code from MESMER is available at https://github.com/MESMER-group/mesmer.
