# Supplementary material for: Showcasing MESMER‐X: Spatially Resolved Emulation of Annual Maximum Temperatures of Earth System Models
Source: Geophys Res Lett. 2022 Aug 30;49(17):e2022GL099012. doi: 10.1029/2022GL099012 (PMC9541273; doi:10.1029/2022GL099012)
Supplement: Supplementary file 1 — Supporting Information S1 [file GRL-49-e2022GL099012-s001.pdf]

AGU PUBLICATIONS

Geophysical Research Letters

Supporting Information for

Showcasing MESMER-X: Spatially resolved emulations of annual maximum temperatures of Earth System Models

Y. Quilcaille[1], L. Gudmundsson[1], L. Beusch[1,*], M. Hauser[1], and S.I. Seneviratne[1]

[1] Institute for Atmospheric and Climate Science, Department of Environmental Systems Science, ETH Zurich, Zurich, Switzerland.

[*] Now at: Federal Office of Meteorology and Climatology, MeteoSwiss, Zurich, Switzerland

Contents of this file

Text S1: Optimization of the first guess for the fit of the GEV with covariates
Text S2: Additional emulator configurations
Figures S1 to S7: Emulator configurations evaluated over different scenarios
Figures S8 to S25: Maps of the CRPSS under different ESMs
Figures S26 to S43: Examples of emulations under different ESMs
Table S1: ESMs, scenarios and ensemble members used

Introduction

This supplementary material provides additional information about the emulation of climate extremes using MESMER-X. The text S1 details how the first guess for the fit of distributions is optimized. The text S2 describes the additional emulator configurations of figures S1-S7. The figures S1-S7 extends the section 4.1, with focus on specific scenarios. The figures S8-S25 extend the section 4.2, illustrating the performances of the emulator under the available ESMs.

Text S1: Optimization of the first guess for the fit of the GEV with covariates

In section 3.1 of the main text of this paper, we describe how a distribution is fitted for the climate extreme, using covariates on parameters. As written in section 3.1, $\Delta X_{s,t}$ corresponds to the sample of the climate extreme and $\boldsymbol{\Delta V_{t,k}}$ to the vector of covariates. We assume immediately that we are on a given gridpoint s to drop the index. In this section, we note ΔX_t the full sample of the climate extreme, historical and scenarios together.

The objective is to identify coefficients for the emulator configuration. We illustrate this method with a GEV here of location μ, scale σ and shape ξ. We write in equation (A.1) the objective. The coefficients μ_0, σ_0 and ξ_0 are constant terms. We separate the i coefficients $\mu_{lin,i}$ on linear covariates from the j coefficients $\mu_{other,j}$ on non-linear covariates, for all parameters.

$$\begin{cases} (\mu_0, \dots \mu_{lin,i} \dots, \dots \mu_{other,j} \dots) \\ (\sigma_0, \dots \sigma_{lin,k} \dots, \dots \sigma_{other,l} \dots) \\ (\xi_0, \dots \xi_{lin,m} \dots, \dots \xi_{other,n} \dots) \end{cases} \quad (A.1)$$

The general idea of this method is to propose a first guess of the constant terms for the location, scale and shape of the distribution using the analytical expressions of the mean, variance and skewness. By optimizing a first evaluation of these constant terms to the observed moments of the distribution, we obtain an optimized first guess.

<u>Step 1:</u>

To begin with, the sample of the climate extreme ΔX_t is detrended using ordinary least squares, and only with the terms on the location that were assumed linear in the emulator configuration. The constant term is noted $\mu_{fg1,0}$, while the coefficients on the i linear terms are written $\mu_{fg,lin,i}$.

<u>Step 2:</u>

From the detrended climate extremes, we deduce the residuals. From these residuals, we calculate the mean M, the variance V and the skewness S of the full sample.

<u>Step 3:</u>

The support of a GEV is defined as shown in equation (A.2). In our data, we observe that the shape is mostly negative, pointing at an upper limit in ΔX_t.

$$\begin{cases} \Delta X_t \in \ [\mu - \sigma/\xi \, , +\infty[\quad when\, \xi > 0 \\ \Delta X_t \in \ ]-\infty, +\infty[\quad when\, \xi = 0 \\ \Delta X_t \in \ ]-\infty, \mu - \sigma/\xi] \quad when\, \xi < 0 \end{cases} \quad (A.2)$$

An initial value ξ_{raw} for the shape is calculated using this support and an ad-hoc value, as shown in equation (A.3). This value will not be the first guess for the shape of the GEV. This ξ_{raw} is meant to ensure that all points of the sample are within the support of the GEV.

$$\xi_{raw} = max\left(-0.25, \frac{V}{M - \max(\Delta X_t)} + 0.1\right) \quad (A.3)$$

Step 4:
We write a first set of coefficients, shown in equation (A.4). The coefficients $\mu_{fg,other,j}$ are written so that the ensuing evolutions would be small compared to the constant. For instance, using notations from Figure 1, the logistic terms are set to $\xi_{\lambda,1}$= 0.1 yr^{-1} and $\xi_{\delta,1} = 0.01\ \xi_0$.

$$\begin{cases} \left(M, \dots \mu_{fg,lin,i} \dots, \dots \mu_{fg,other,j} \dots\right) \\ \left(\sqrt{V}, \dots 0 \dots, \dots \sigma_{fg,other,l} \dots\right) \\ \left(\xi_{raw}, \dots 0 \dots, \dots \xi_{fg,other,n} \dots\right) \end{cases} \quad (A.4)$$

The mean M_{GEV}, the variance V_{GEV} and the skewness S_{GEV} of a GEV of location μ, scale σ and shape ξ can be written as shown in equations (A.5). We write γ as the Euler's constant, Γ as the Gamma function, sgn as the sign function and ζ as the Riemann's zeta function.

$$\begin{cases} M_{GEV} = \begin{cases} \mu + \sigma\,(g_1 - 1)/\xi \quad when\ \xi \neq 0, \xi < 1 \\ \mu + \sigma\gamma \quad when\ \xi = 0 \\ \infty \quad when\ \xi \geq 1 \end{cases} \\ V_{GEV} = \begin{cases} \sigma^2\,(g_2 - g_1^2)/\xi^2 \quad when\ \xi \neq 0, \xi < 1/2 \\ \sigma^2\,\pi^2/6 \quad when\ \xi = 0 \\ \infty \quad when\ \xi \geq 1/2 \end{cases} \\ S_{GEV} = \begin{cases} sgn(\xi)\dfrac{g_3 - 3g_2g_1 + 2g_1^3}{(g_2 - g_1^2)^{\frac{3}{2}}} \quad when\ \xi \neq 0, \xi < \dfrac{1}{3} \\ 12\sqrt{6}\,\zeta(3)/\pi^3 \quad when\ \xi = 0 \end{cases} \\ g_k = \Gamma(1 - k\xi) \end{cases} \quad (A.5)$$

We optimize now the constant coefficients (μ_c, σ_c, ξ_c) with starting values $\left(M, \sqrt{V}, \xi_{raw}\right)$ from (A.4), by minimization of the differences to the moments of the GEV deduced from (A.5). This process is illustrated in equation (A.6), and the solution is noted $\left(\mu_{fg2,0}, \sigma_{fg,0}, \xi_{fg,0}\right)$.

$$\left(\mu_{fg2,0}, \sigma_{fg,0}, \xi_{fg,0}\right) = \ min_{(\mu_c,\sigma_c,\xi_c)}^{constraints} \begin{pmatrix} (M_{GEV}(\mu_c, \sigma_c, \xi_c) - M)^2 \\ +(V_{GEV}(\mu_c, \sigma_c, \xi_c) - V)^2 \\ +(S_{GEV}(\mu_c, \sigma_c, \xi_c) - S)^2 \end{pmatrix} \quad (A.6)$$

Equation (A.6) shows that the minimization is performed with constraints. At every step of the minimization, a set of coefficients (μ_c, σ_c, ξ_c) is tried to reach the solution $\left(\mu_{fg2,0}, \sigma_{fg,0}, \xi_{fg,0}\right)$. At each of these steps, the evolutions over time of the parameters (μ_t, σ_t, ξ_t) of the GEV are computed. To do so, the covariates are used along the coefficients from equation (A.4), although values $\left(M, \sqrt{V}, \xi_{raw}\right)$ are replaced by the current values $\left(\mu_{fg1,0} + \mu_c, \sigma_c, \xi_c\right)$. We pinpoint that the actual mean for the calculation of the evolution of the coefficients was $\mu_{fg1,0} + \mu_c$, not only μ_c. This is due to the dependency of the mean of the GEV to its scale and shape, as shown in equation (A.5), and the linear detrend used in step 1.
The computation of the evolutions of the parameters allow the verification of conditions, as shown in equation (A.7). The first condition verifies that the sample falls within the support of the current tested GEV, and is a direct consequence of equation (A.2). The second condition is meant to avoid problematic values on the shape. The low and high

thresholds on the shape were respectively set to $-\infty$ and $^1/_3$, to avoid an infinite skewness, as shown in equation (A.5). The third condition simply answers to obvious mathematical and physical grounds. The fourth condition is meant to avoid spurious evolutions of coefficients in ill-defined emulator configurations, causing a trend in coefficients, almost compensating in the evolutions of parameters. This second low threshold were set to -2, this value were observed to provide good results. The last condition actually corresponds to other mathematical conditions on coefficients, such as the time constant in logistic evolutions that are meant to be positive.

$$\begin{cases} \begin{cases} \Delta X_t \in [\mu_t - \sigma_t/\xi_t\,, +\infty[& when\ \xi_t > 0 \\ \Delta X_t \in\ ]-\infty, \mu_t - \sigma_t/\xi_t] & when\ \xi_t < 0 \end{cases} \\ \xi_t \in [\xi_{threshold,low}, \xi_{threshold,high}] \\ \sigma_t > 0 \\ \xi_c > \xi_{threshold,low,2} \\ \xi_\lambda > 0, \ldots \end{cases} \quad (A.7)$$

<u>Step 5:</u>

Thanks to the former optimization, better values for the constant terms have been found. By feeding the result of (A.6) in (A.4), we calculate the negative log likelihood of the current solution, a first optimized first guess.
Then we repeat step 4, although by removing the term on the mean. The second optimized first guess is then used to calculate the negative log likelihood.
We deduce the first guess by taking the one with the lower negative log likelihood.
Equation (A.8) shows the optimal first guess used for the fit of the distribution from section 3.1. We pinpoint that the conditions (A.7) are used as well during the fit of the distribution.

$$\begin{cases} (\mu_{fg1,0} + \mu_{fg2,0}, \ldots \mu_{fg,lin,i} \cdots, \ldots \mu_{fg,other,j} \cdots) \\ (\sigma_{fg,0}, \ldots 0 \ldots, \ldots \sigma_{fg,other,l} \cdots) \\ (\xi_{fg,0}, \ldots 0 \ldots, \ldots \xi_{fg,other,n} \cdots) \end{cases} \quad (A.8)$$

Text S2: Additional emulator configurations

Figures S.1 to S.7 show additional emulator configurations. We use an additional driver, ΔH^{GT}, the global trend of the anomaly in HFDS, to disentangle contributions with different timescales.

The figures S.1 to S.7 show that ΔH^{GT} does not bring the desired improvement: the differences in transient and equilibrium TXx appear mostly at the end of low-warming scenarios. Using the extensions of scenarios up to 2300 may help the algorithm in seizing this signal.

Evolutions on the shape of the distribution have been investigated as well through the shape parameters. Some processes may affect the local dominant processes driving annual maximum temperatures. Linear and logistic evolutions have been tried, respectively for simplicity or for transition between two regimes. These evolutions did not bring the expected improvements. Bigger samples may help emulating such features.

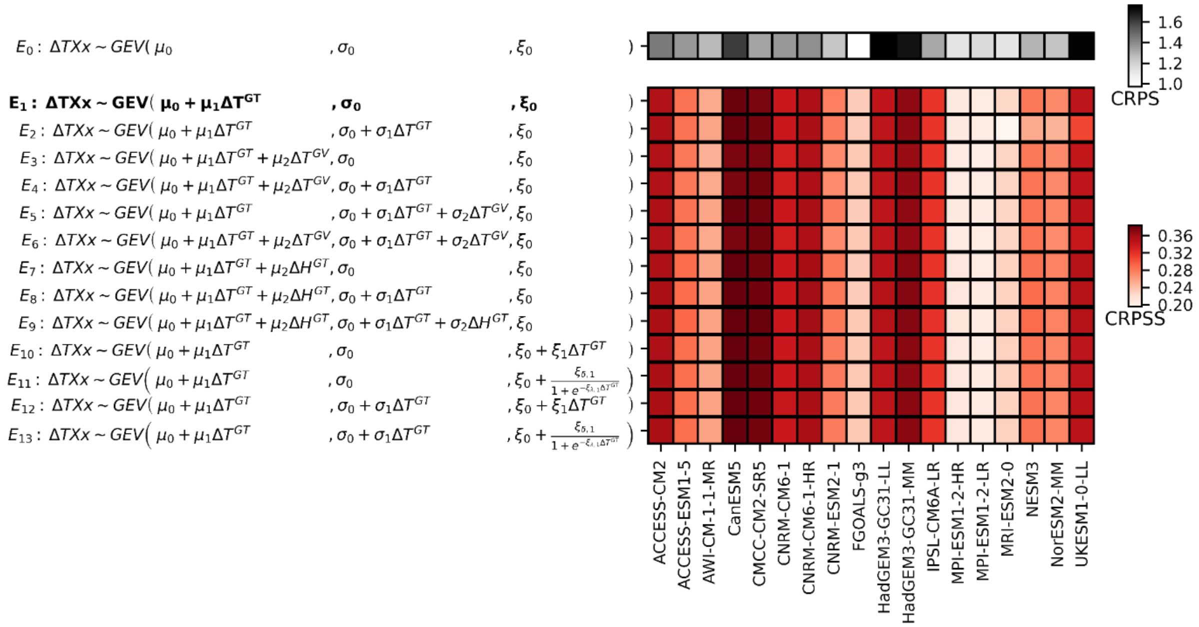

Figure S.1. Emulator configurations over historical and all scenarios. The first row shows the CRPS (lower is better) for E_0 used as a reference. On the following rows, the CRPSS (higher is better) with reference to the emulator configuration E_0 show the respective global performance of the different emulator configurations for different ESMs.

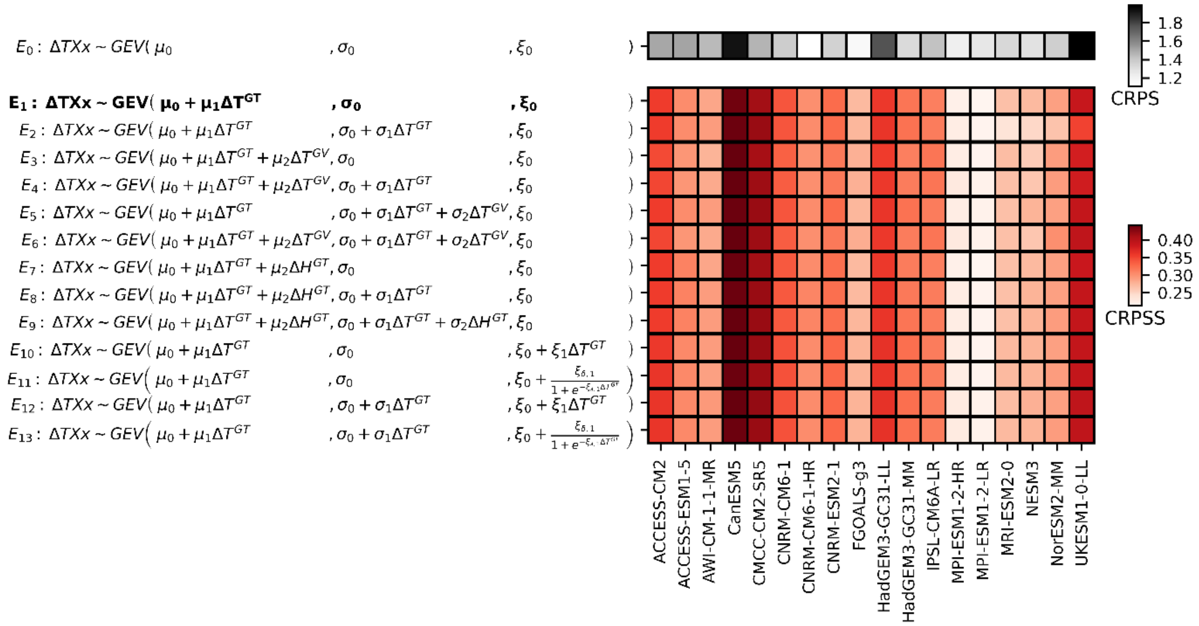

Figure S.2. Same as Figure S.1, with training over all available scenarios, but evaluation solely over the historical (1850-2014).

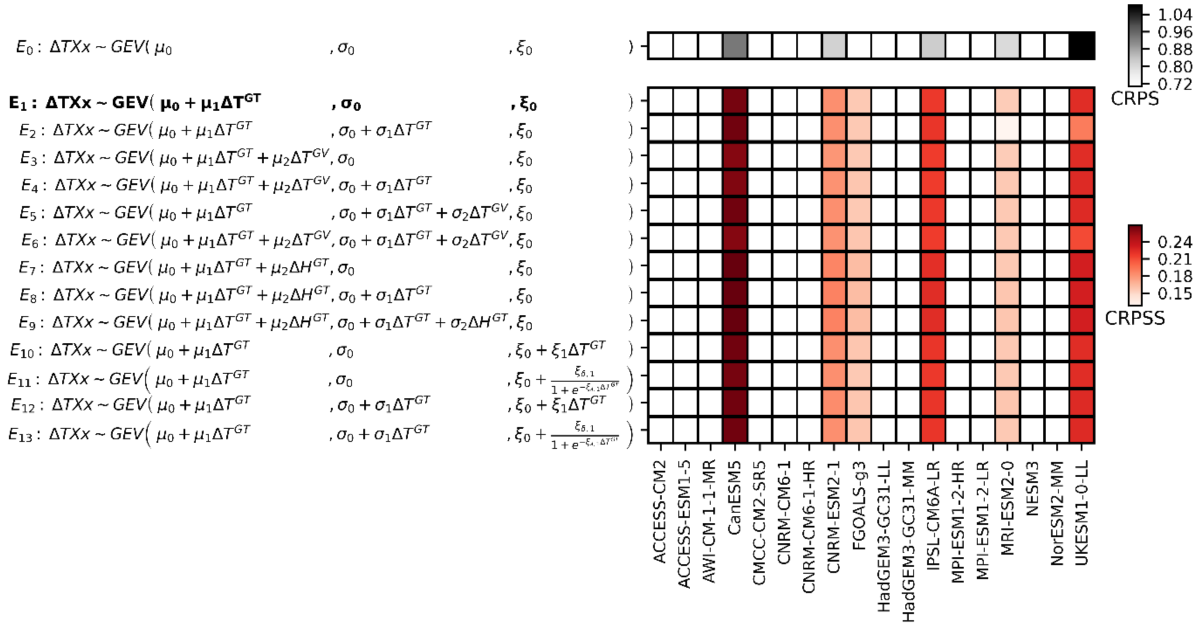

Figure S.3. Same as Figure S.1, with training over all available scenarios, but evaluation solely over the *ssp119* (2015-2100).

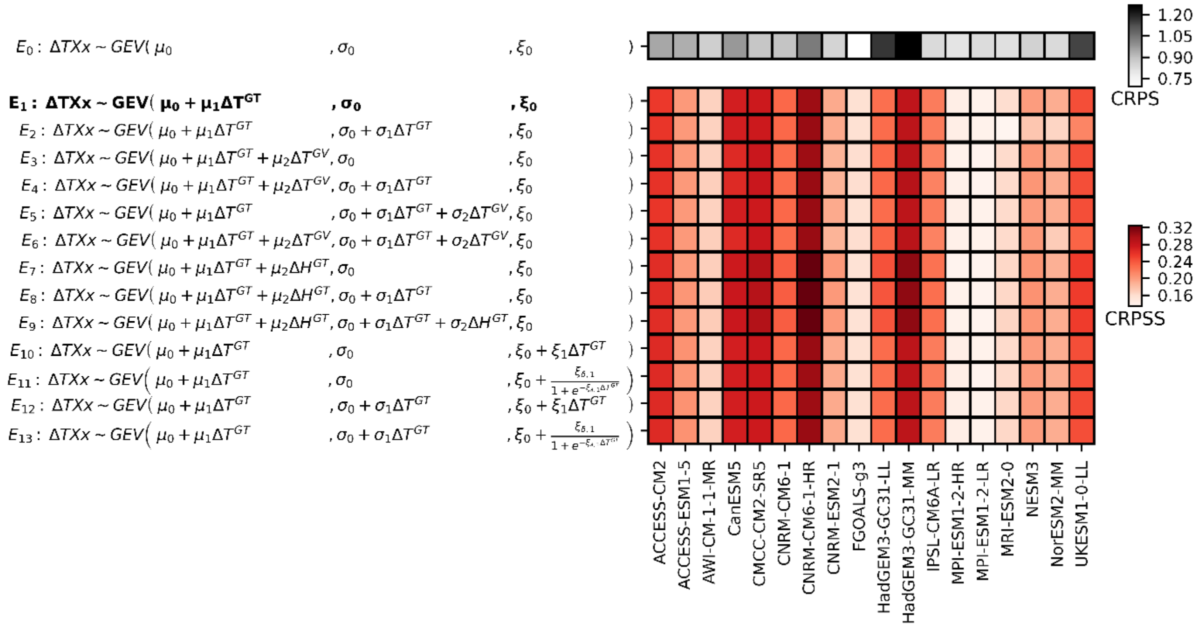

Figure S.4. Same as Figure S.1, with training over all available scenarios, but evaluation solely over the *ssp126* (2015-2100).

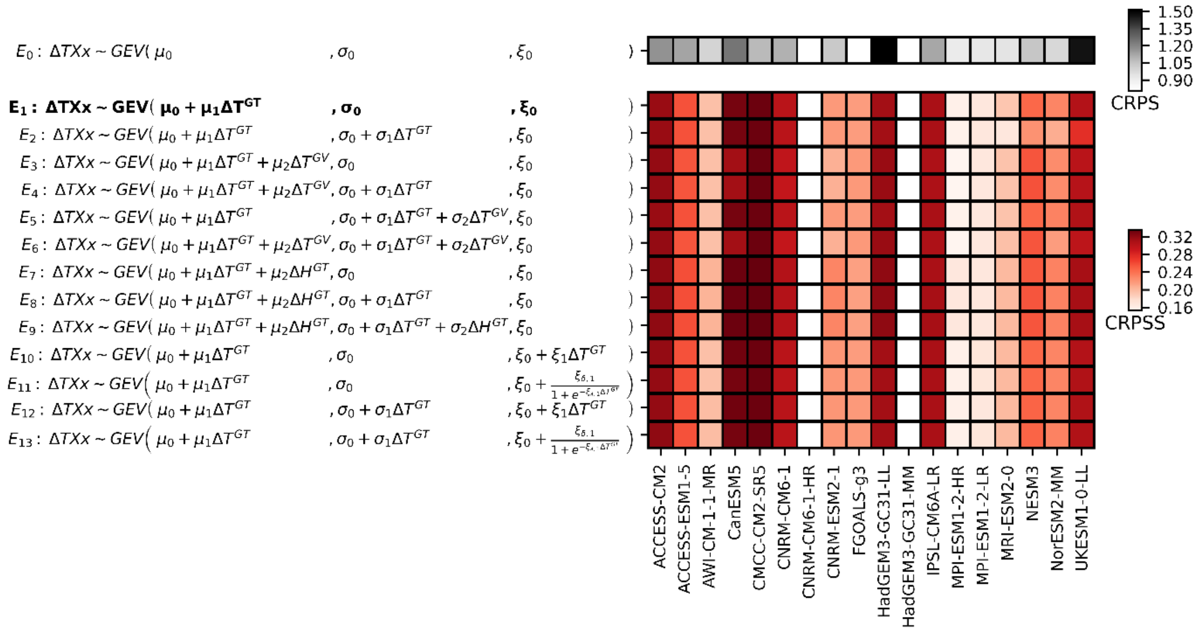

Figure S.5. Same as Figure S.1, with training over all available scenarios, but evaluation solely over the *ssp245* (2015-2100).

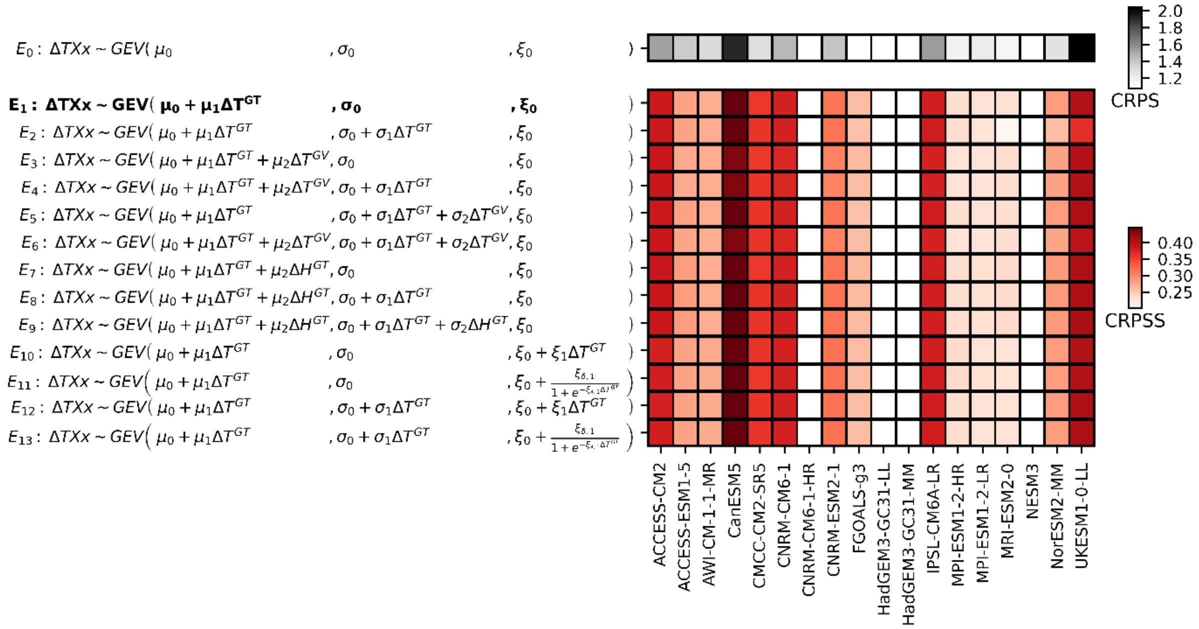

Figure S.6. Same as Figure S.1, with training over all available scenarios, but evaluation solely over the *ssp370* (2015-2100).

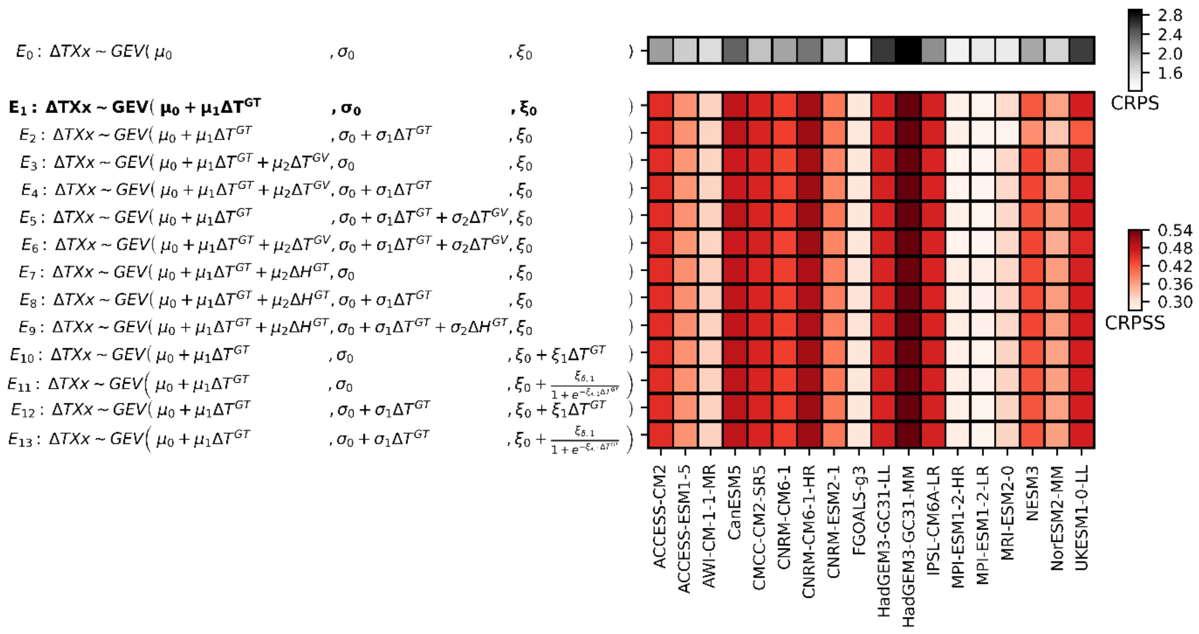

Figure S.7. Same as Figure S.1, with training over all available scenarios, but evaluation solely over the *ssp585* (2015-2100).

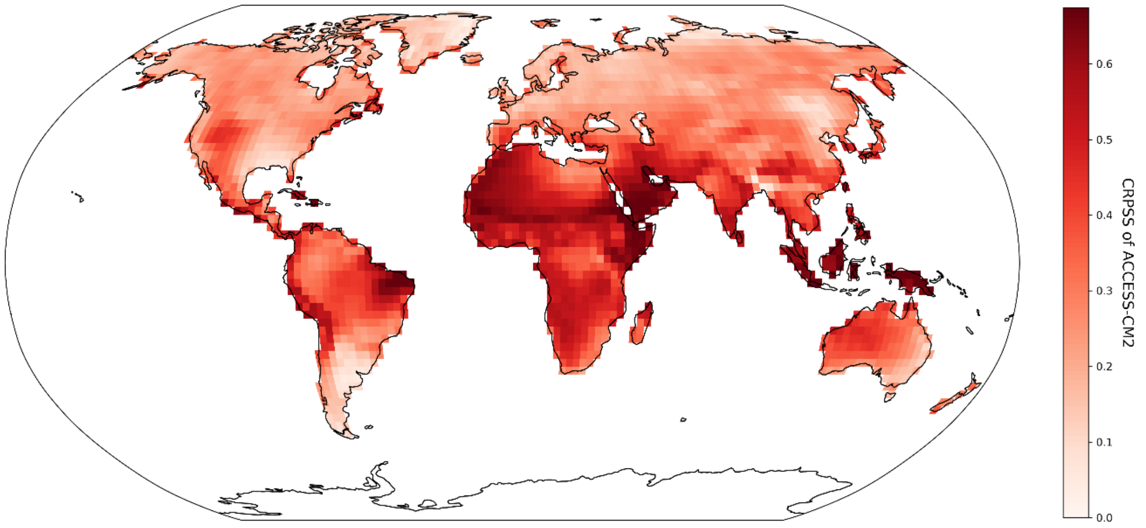

Figure S.8. Map of the error metric CRPSS over historical and available scenarios for the emulator configuration E1 and ACCESS-CM2.

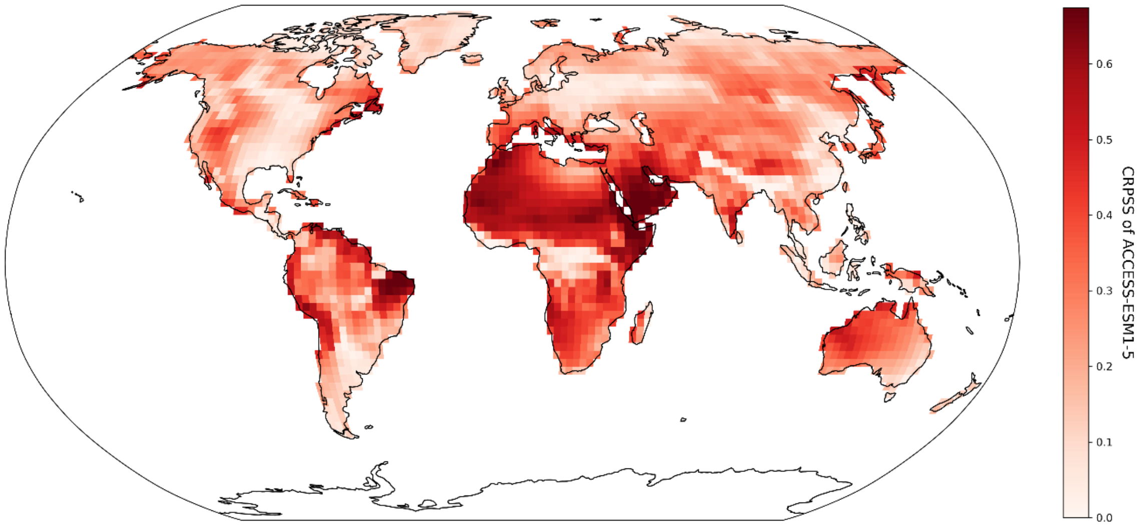

Figure S.9. Same as Figure S.8, but for ACCESS-ESM1-5.

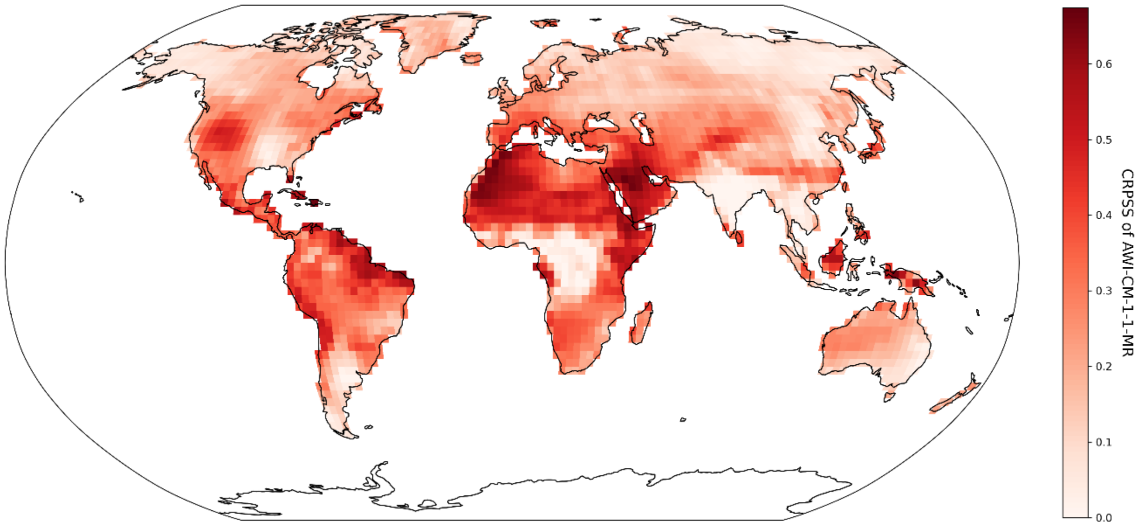

Figure S.10. Same as Figure S.8, but for AWI-CM1-1-MR.

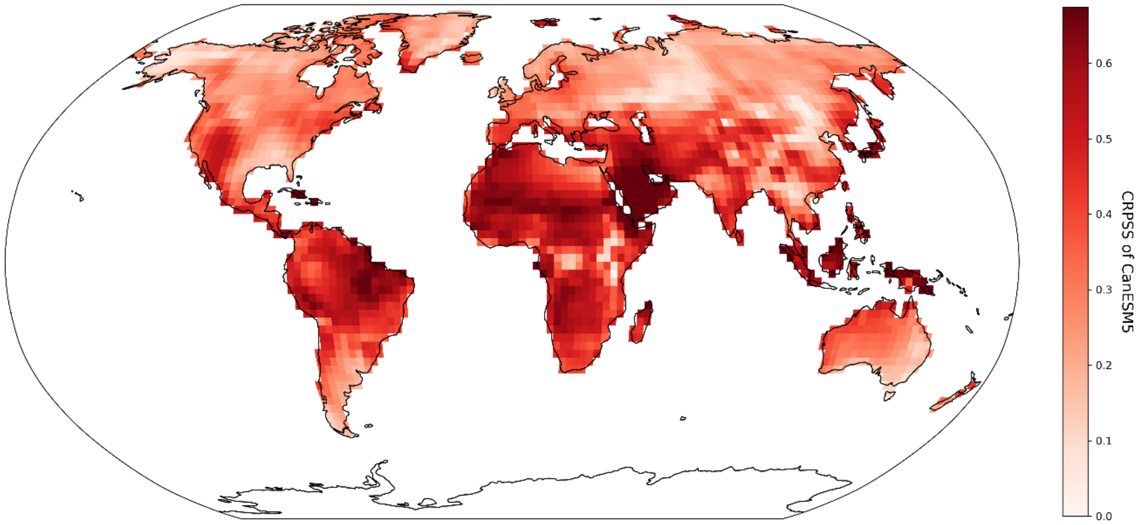

Figure S.11. Same as Figure S.8, but for CanESM5.

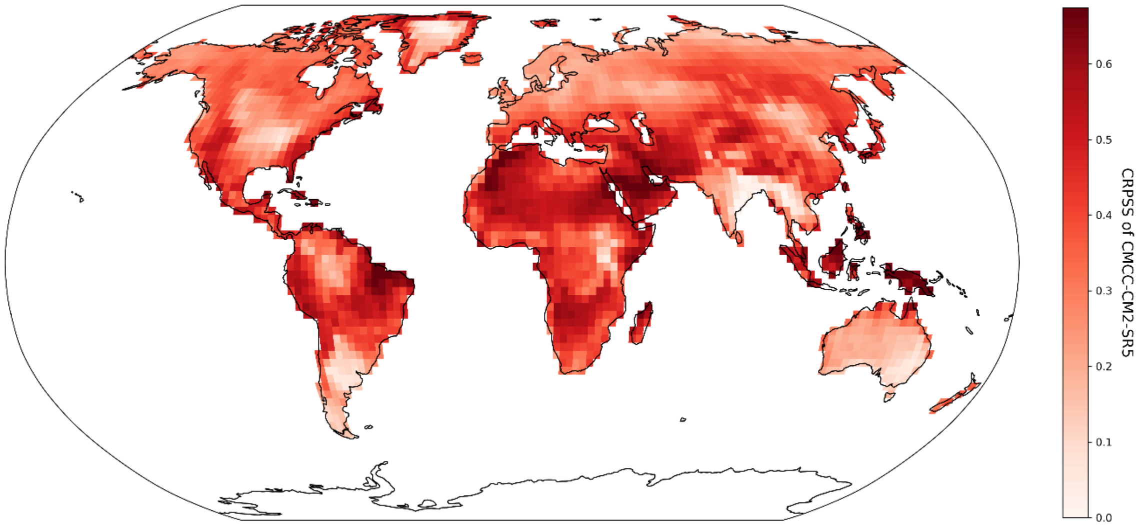

Figure S.12. Same as Figure S.8, but for CMCC-CM2-SR5.

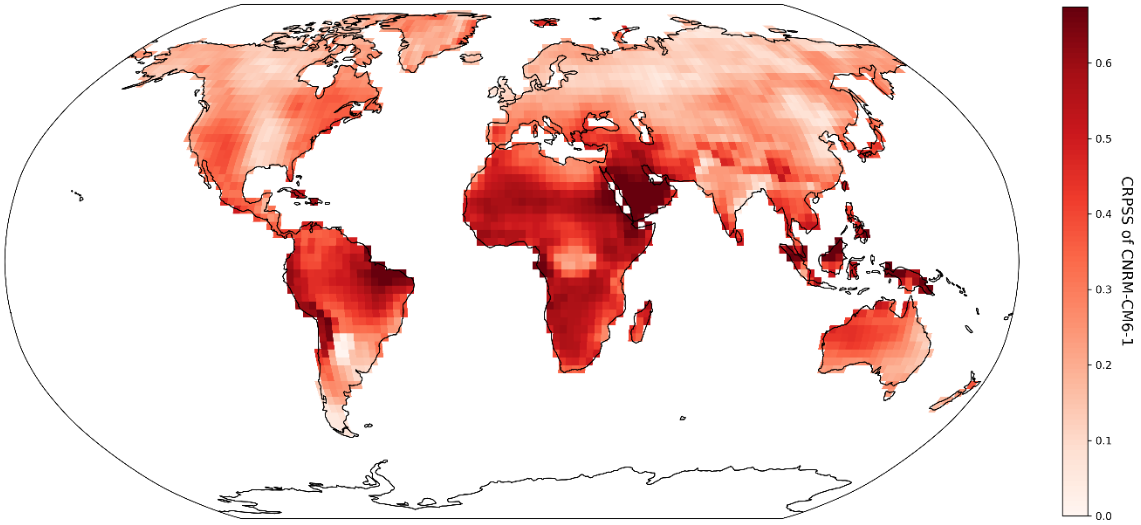

Figure S.13. Same as Figure S.8, but for CNRM-CM6-1.

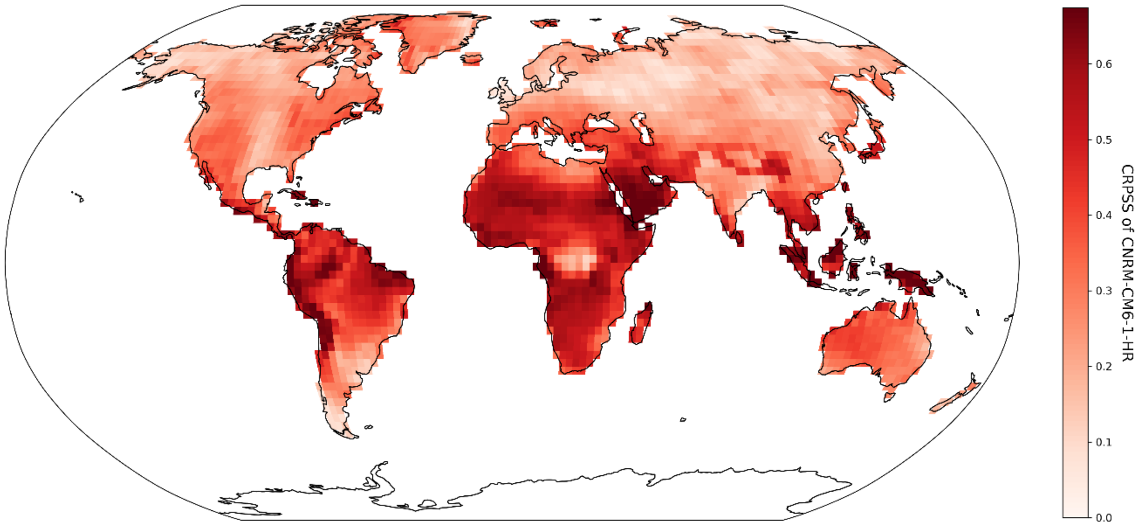

Figure S.14. Same as Figure S.8, but for CNRM-CM6-1-HR.

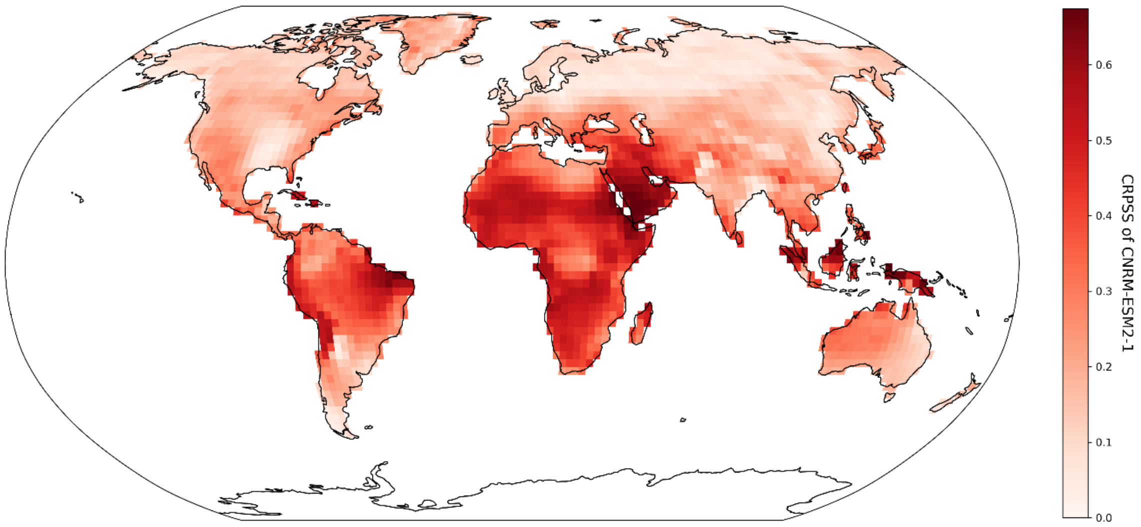

Figure S.15. Same as Figure S.8, but for CNRM-ESM2-1.

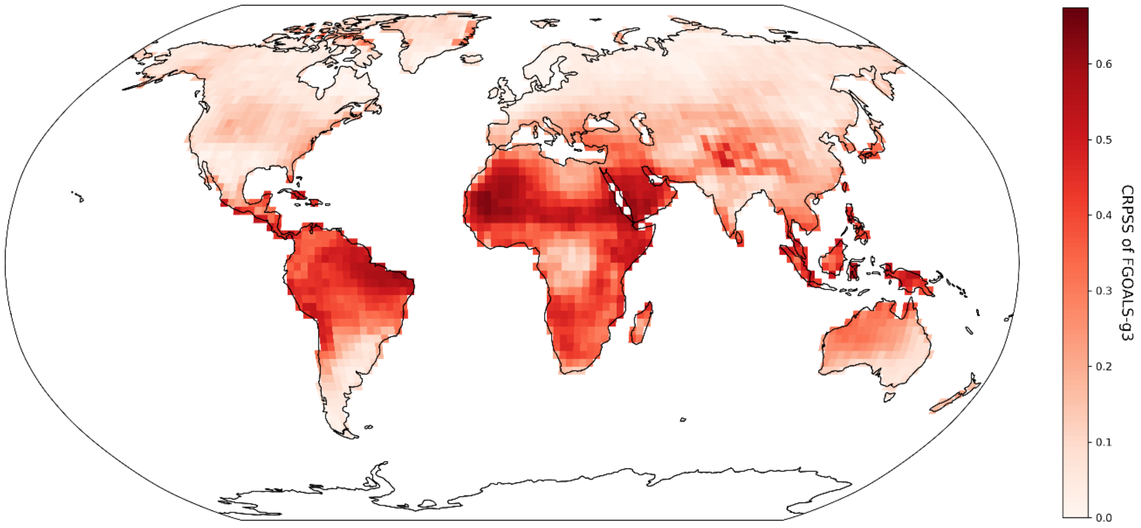

Figure S.16. Same as Figure S.8, but for FGOALS-g3.

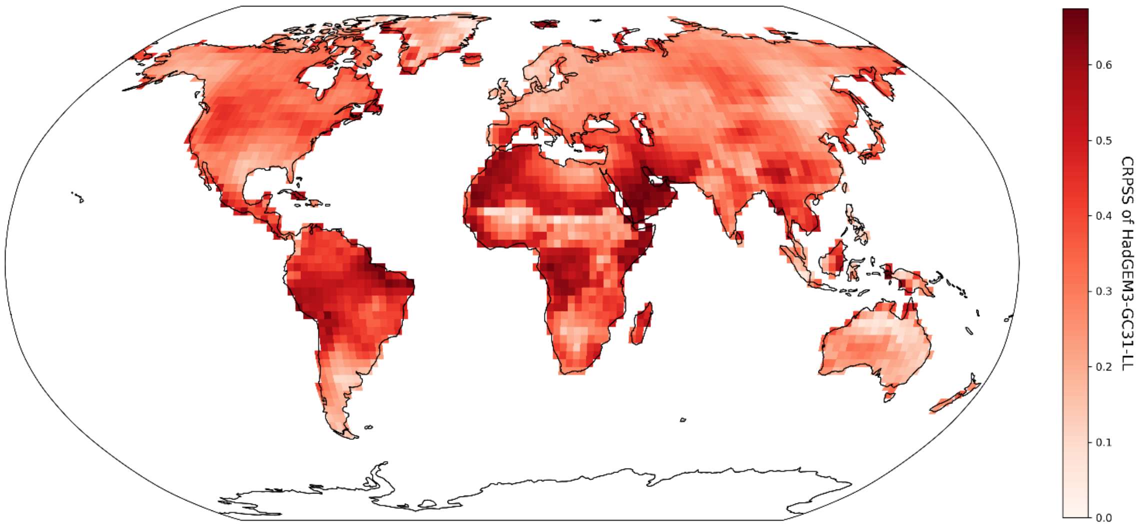

Figure S.17. Same as Figure S.8, but for HadGEM3-GC31-LL.

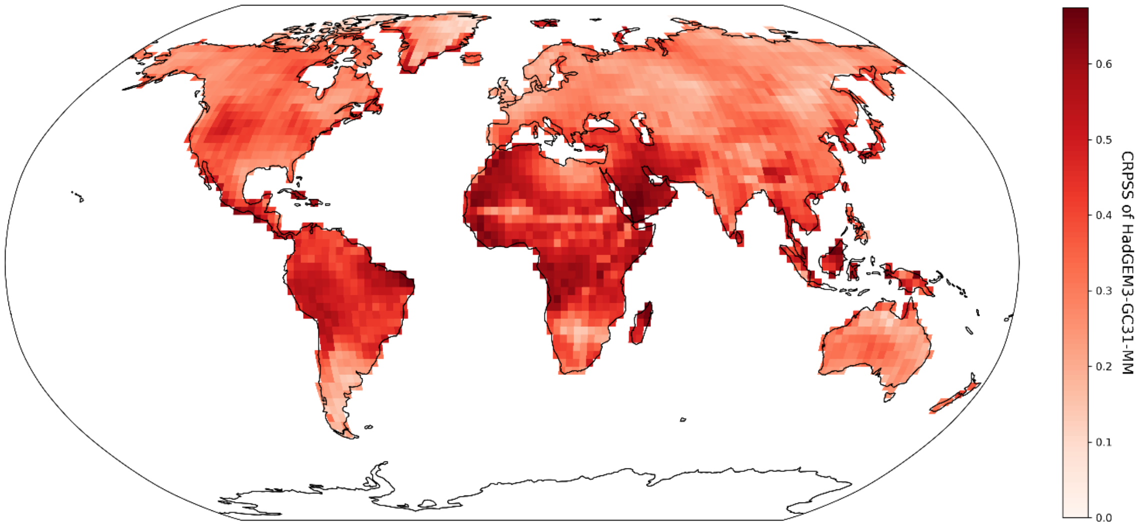

Figure S.18. Same as Figure S.8, but for HadGEM3-GC31-MM.

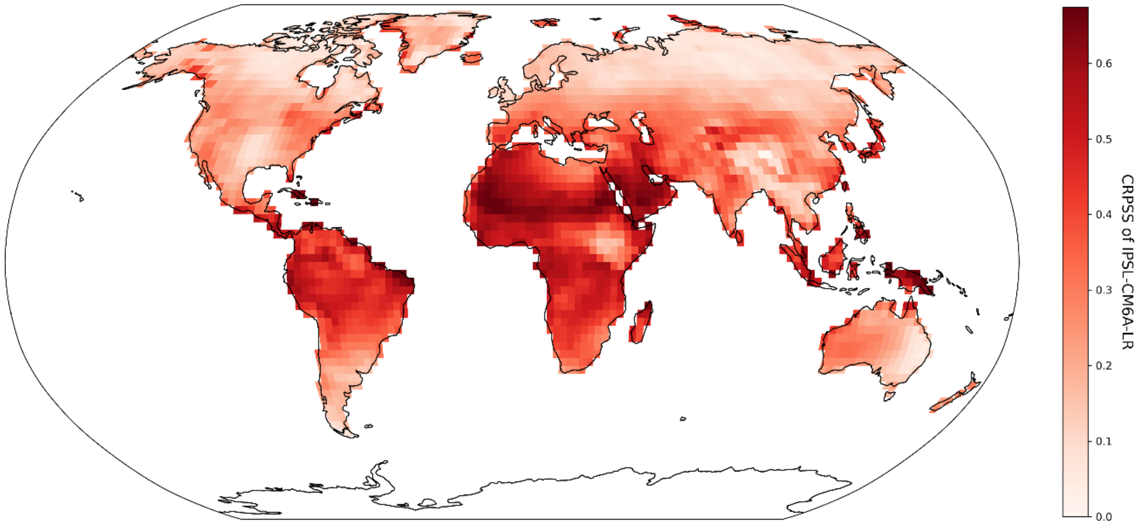

Figure S.19. Same as Figure S.8, but for IPSL-CM6A-LR.

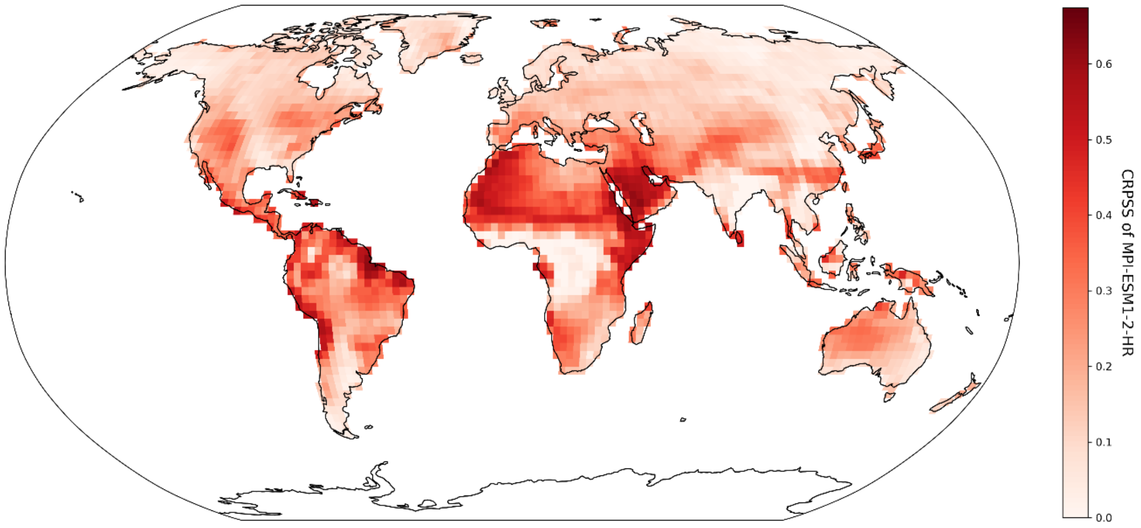

Figure S.20. Same as Figure S.8, but for MPI-ESM1-2-HR.

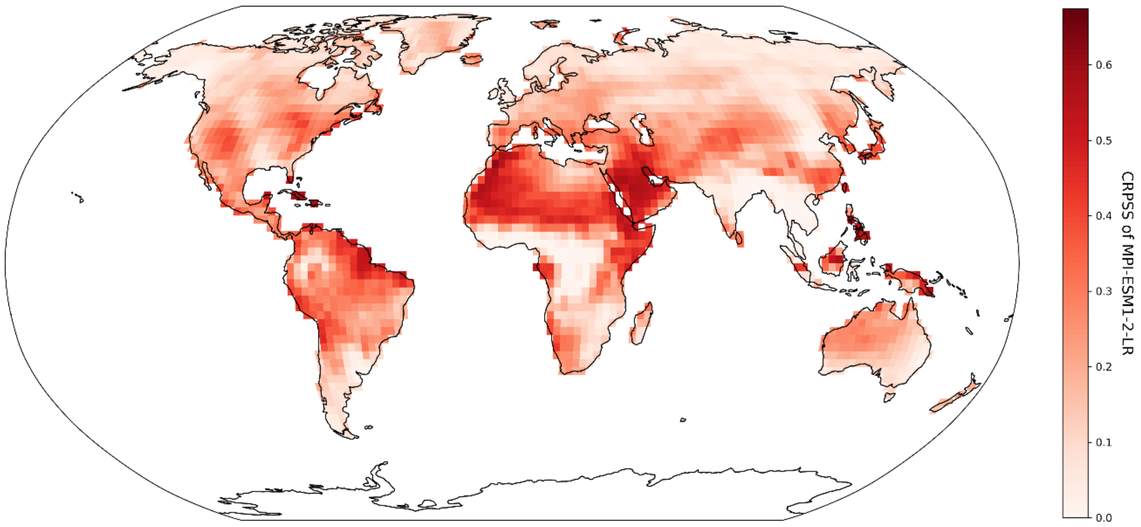

Figure S.21. Same as Figure S.8, but for MPI-ESM1-2-LR.

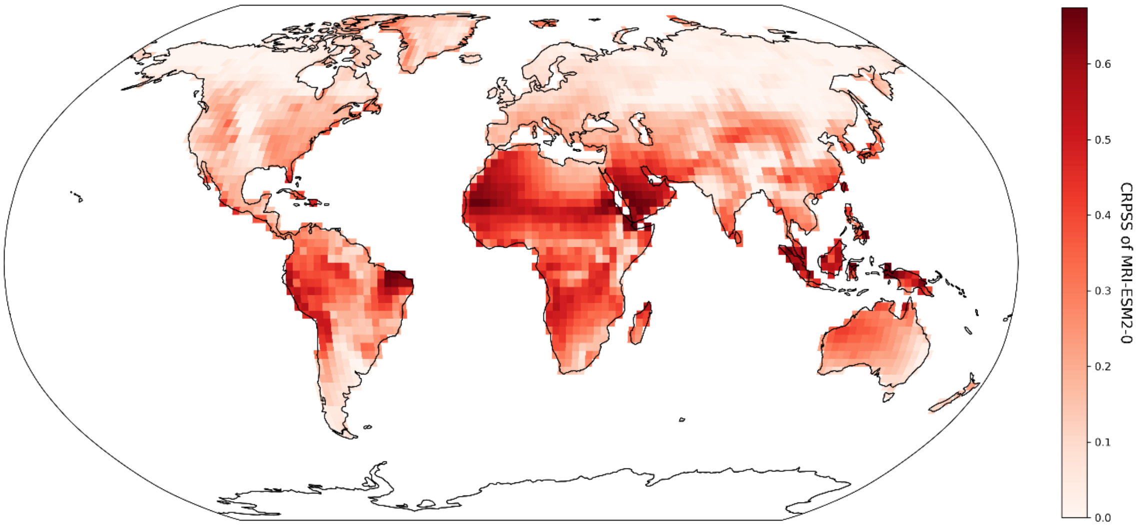

Figure S.22. Same as Figure S.8, but for MRI-ESM2-0.

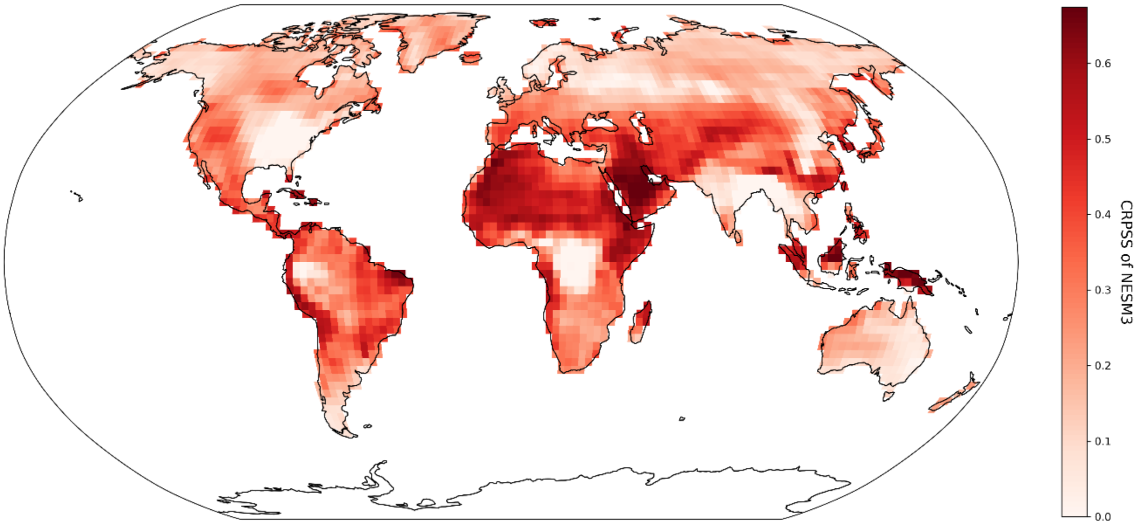

Figure S.23. Same as Figure S.8, but for NESM3.

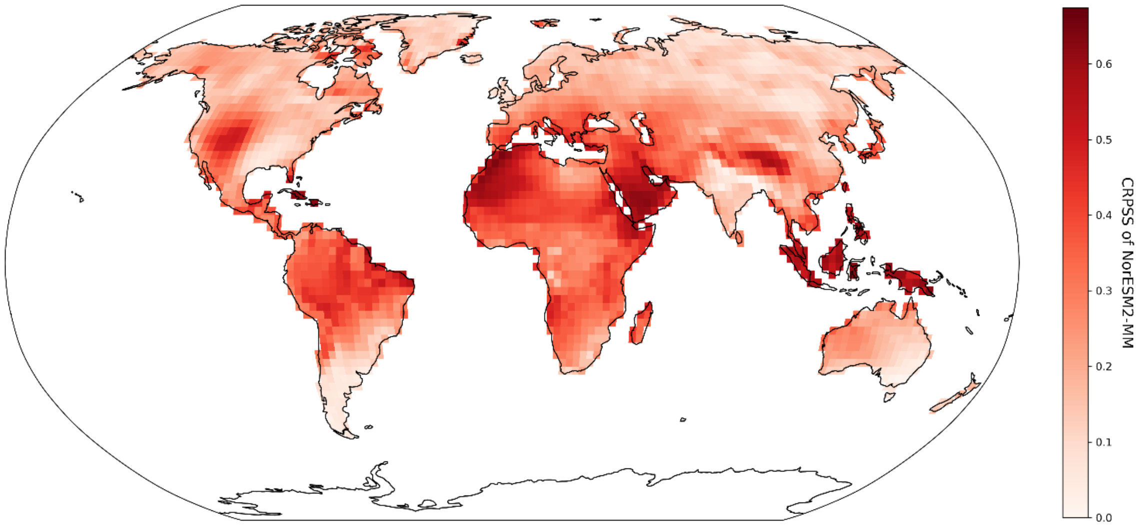

Figure S.24. Same as Figure S.8, but for NorESM2-MM.

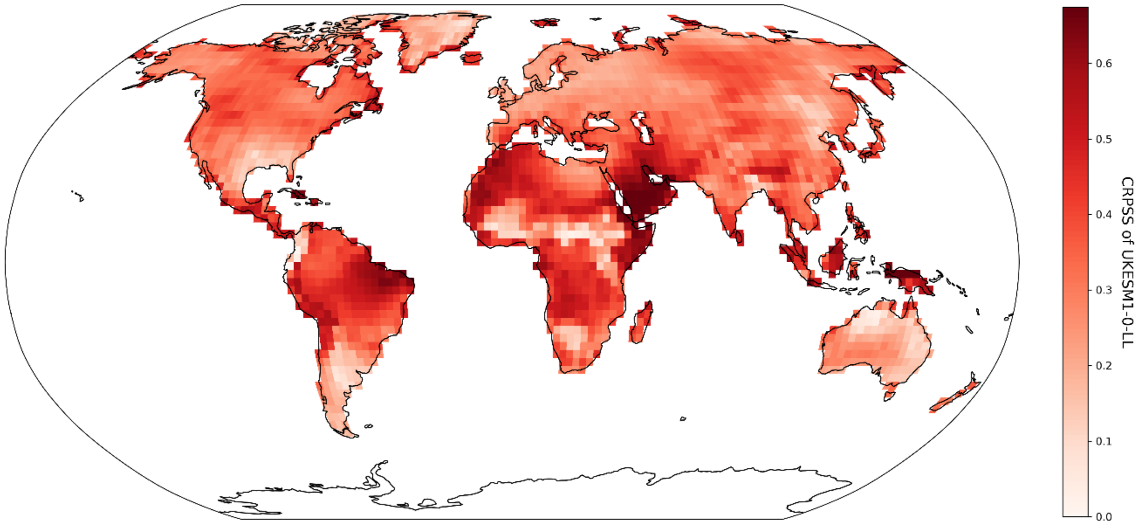

Figure S.25. Same as Figure S.8, but for UKESM1-0-LL.

Examples of emulations under each ESM:

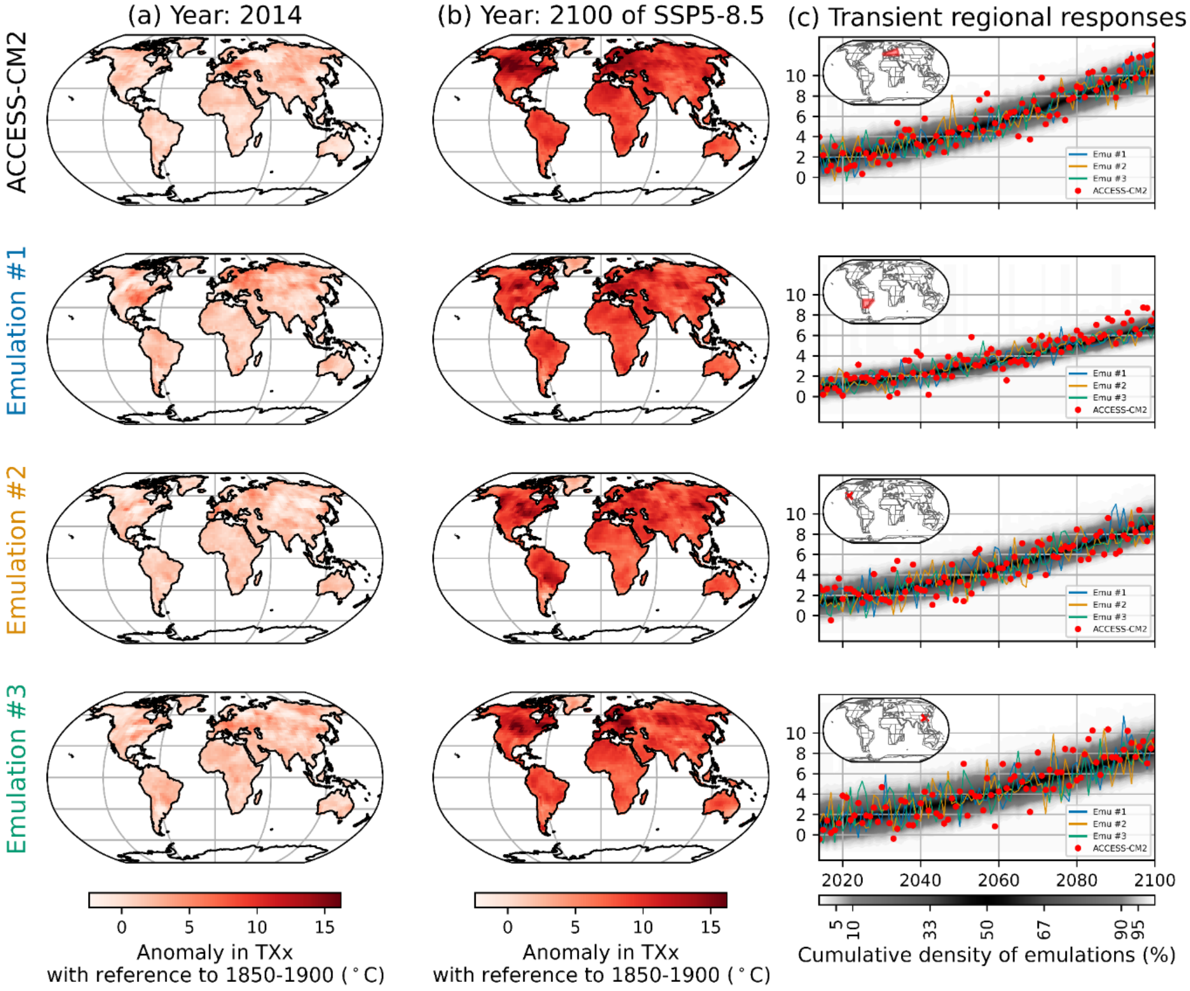

Figure S.26. Example of emulations for ACCESS-CM2 and three of its emulations in 2014 and 2100, in columns (a) and (b), respectively. The transient regional response from 2014 to 2100 are shown in column (c) for selected regions and grid points. It features the values from ACCESS-CM2, the same three emulations shown in maps and the density of the 1000 emulations drawn for this emulator configuration.

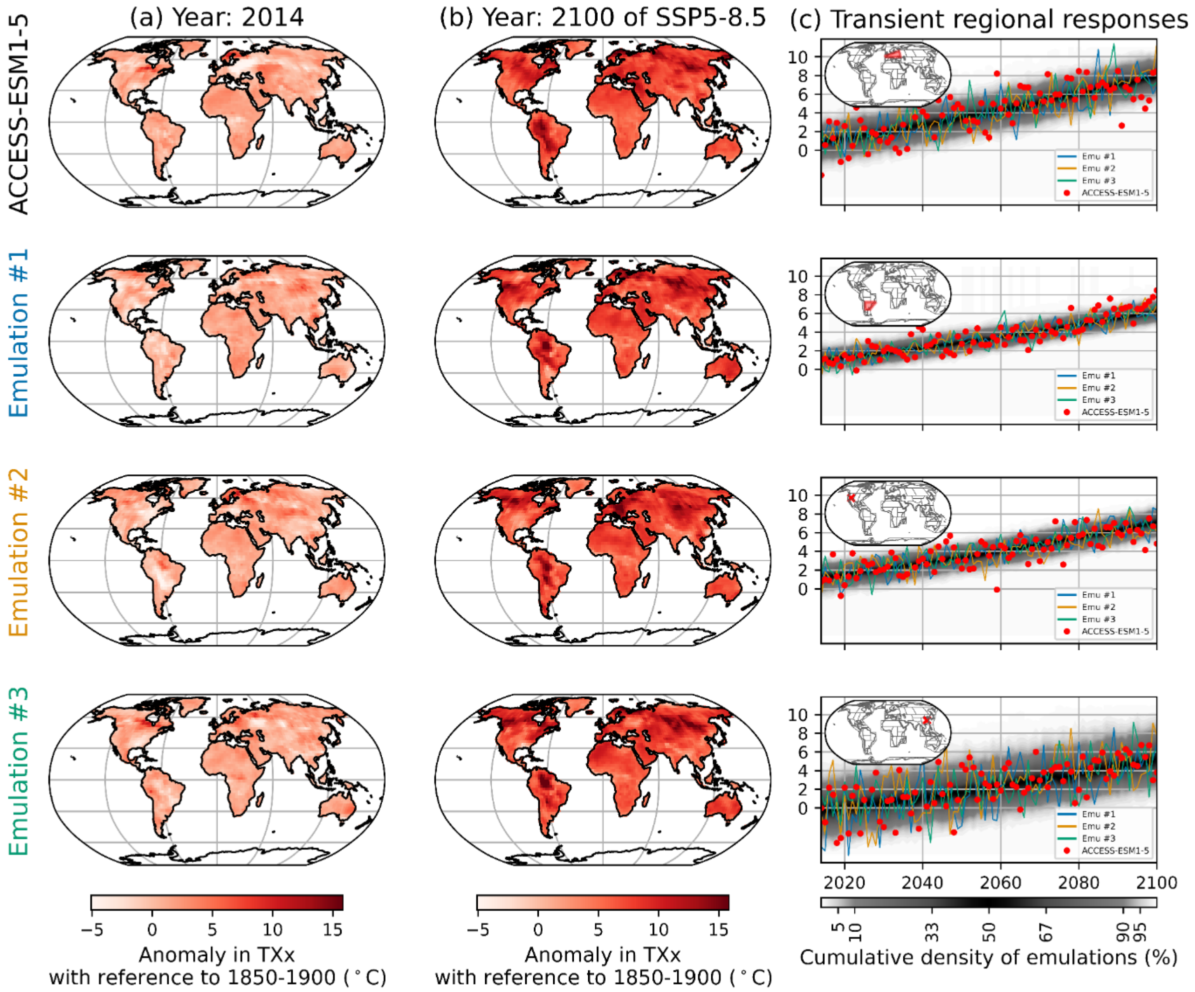

Figure S.27. Same as Figure S.8, but with ACCESS-ESM1-5. Note that the scales are adapted to this ESM.

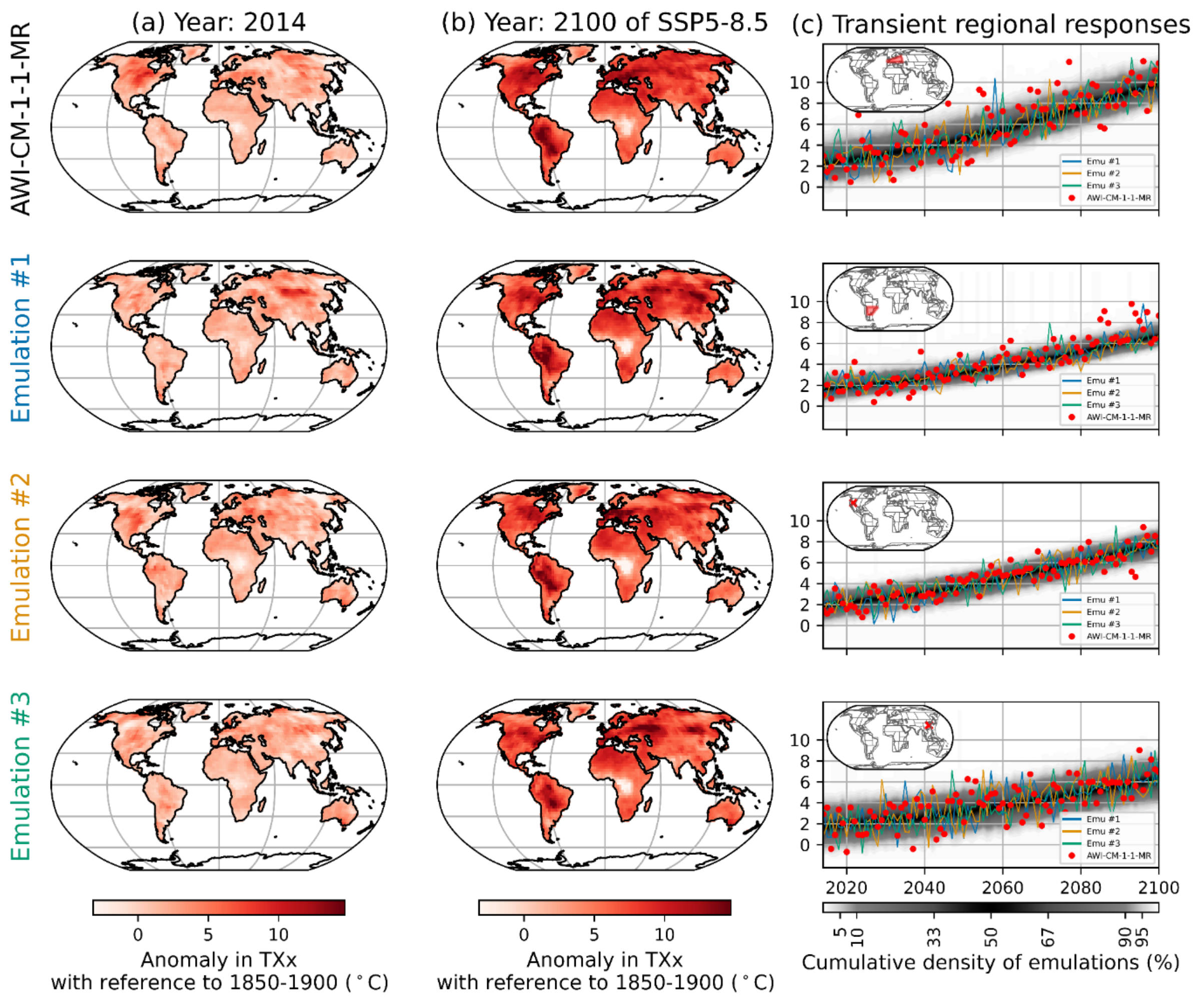

Figure S.28. Same as Figure S.8, but with AWI-CM-1-1-MR. Note that the scales are adapted to this ESM.

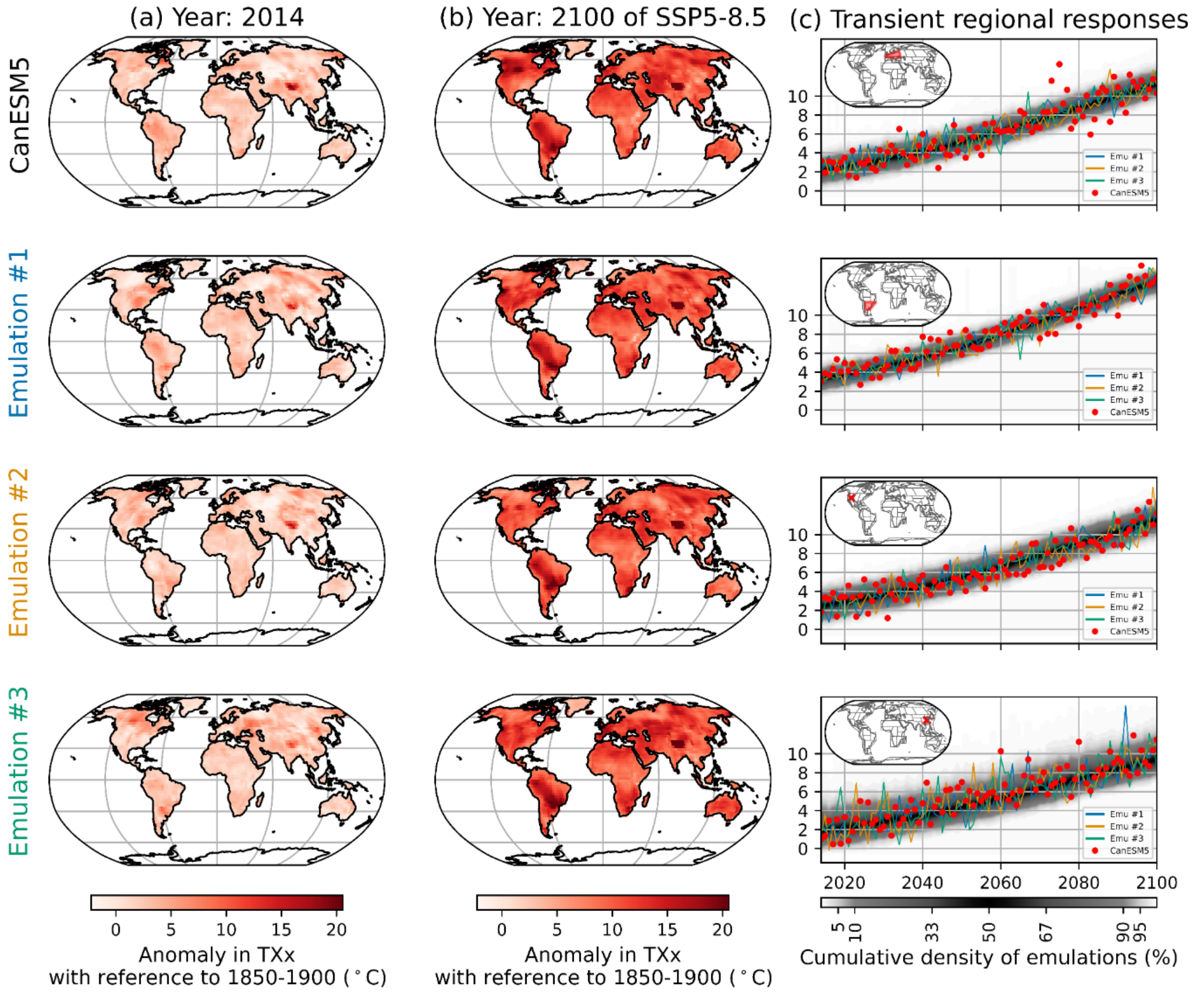

Figure S.29. Same as Figure S.8, but with CanESM5. Note that the scales are adapted to this ESM.

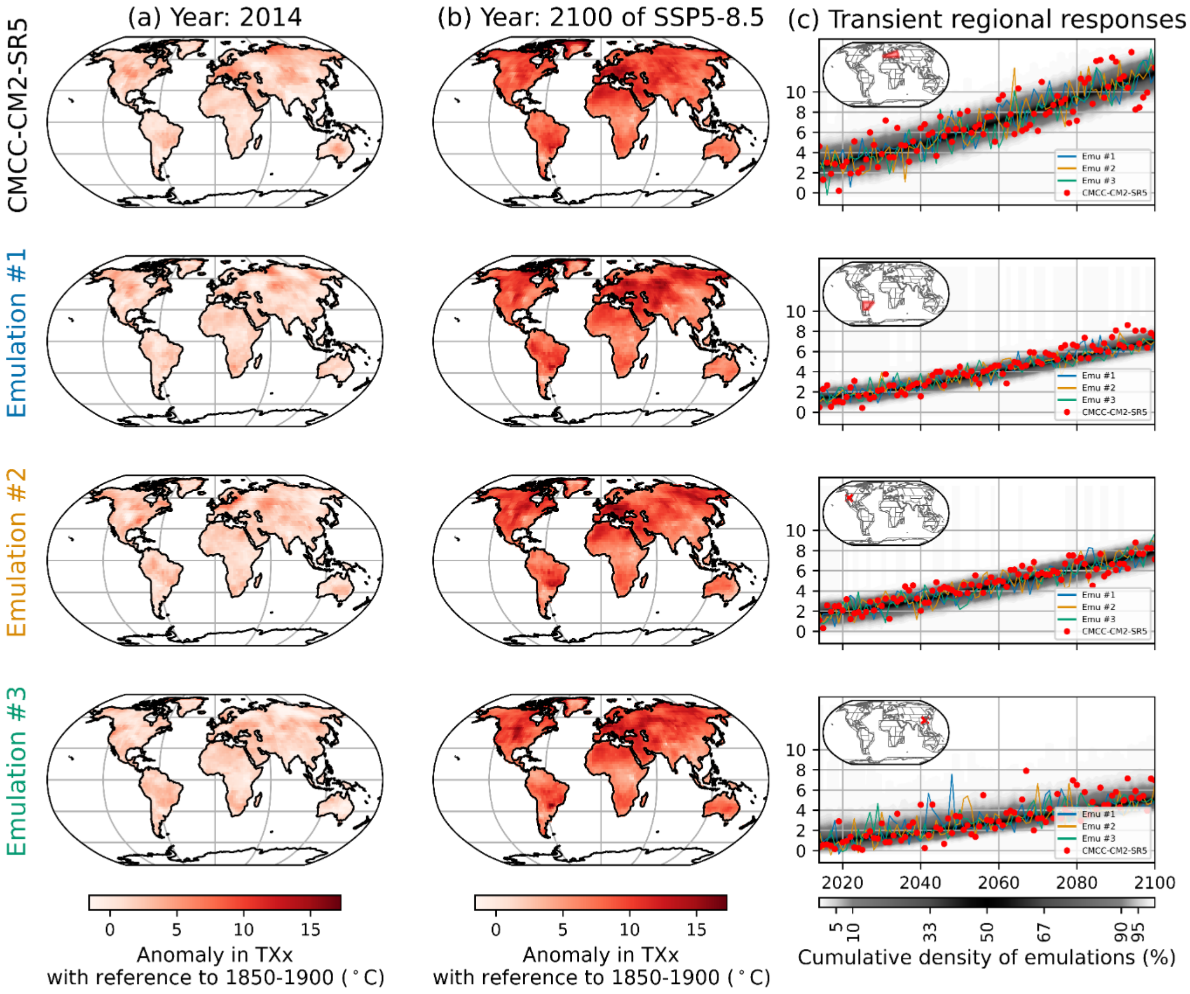

Figure S.30. Same as Figure S.8, but with CMCC-CM2-SR5. Note that the scales are adapted to this ESM.

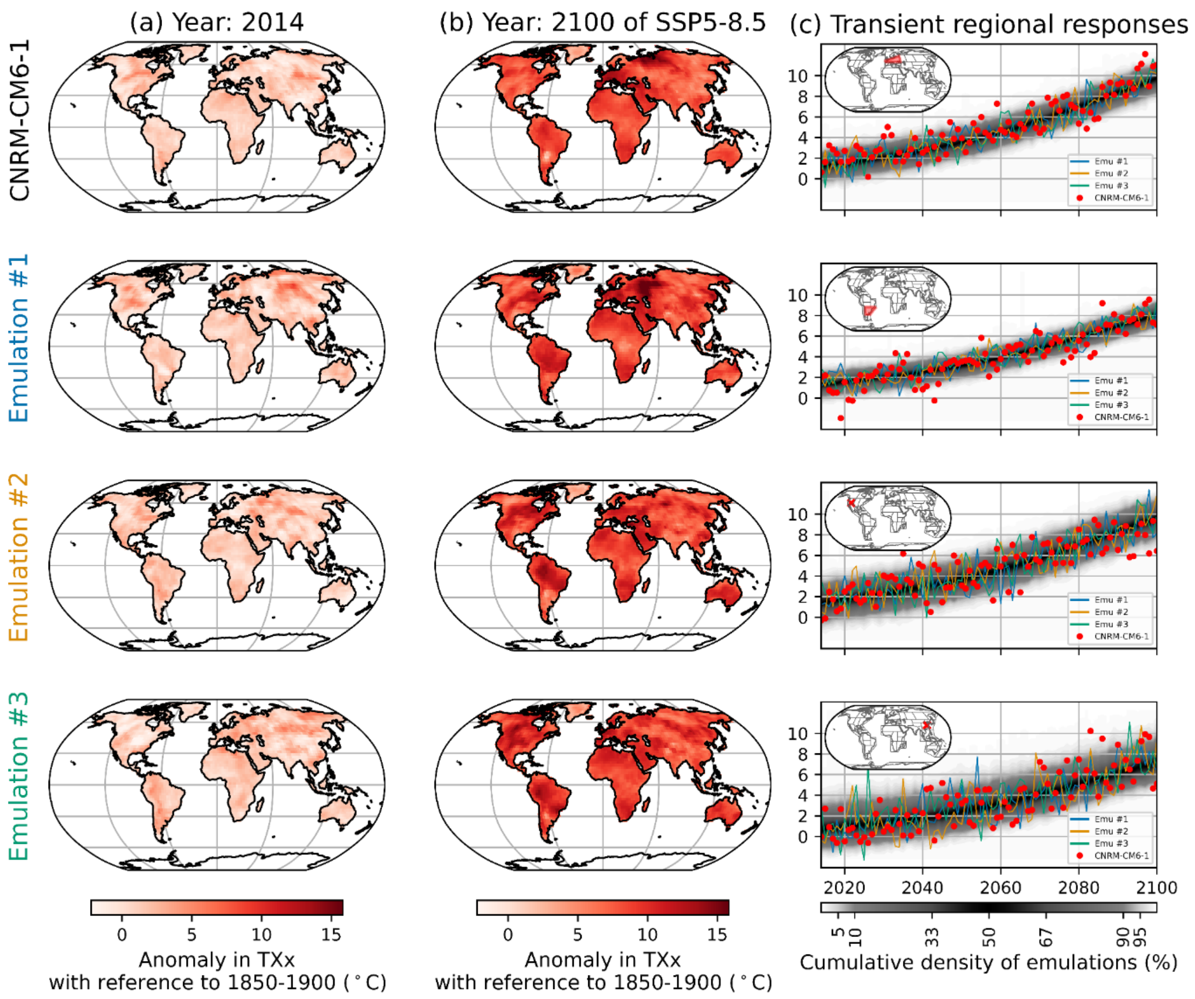

Figure S.31. Same as Figure S.8, but with CNRM-CM6-1. Note that the scales are adapted to this ESM.

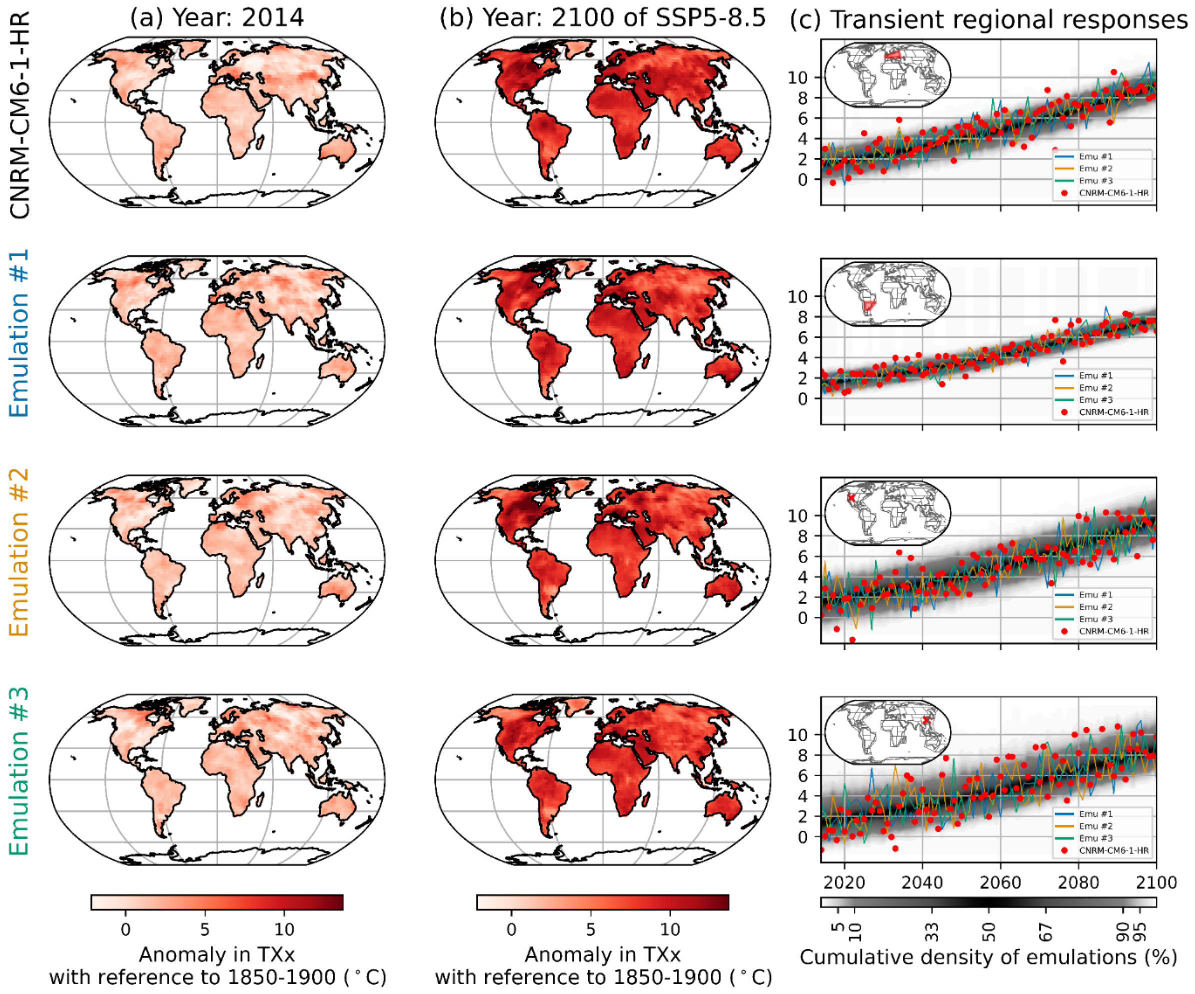

Figure S.32. Same as Figure S.8, but with CNRM-CM6-1-HR. Note that the scales are adapted to this ESM.

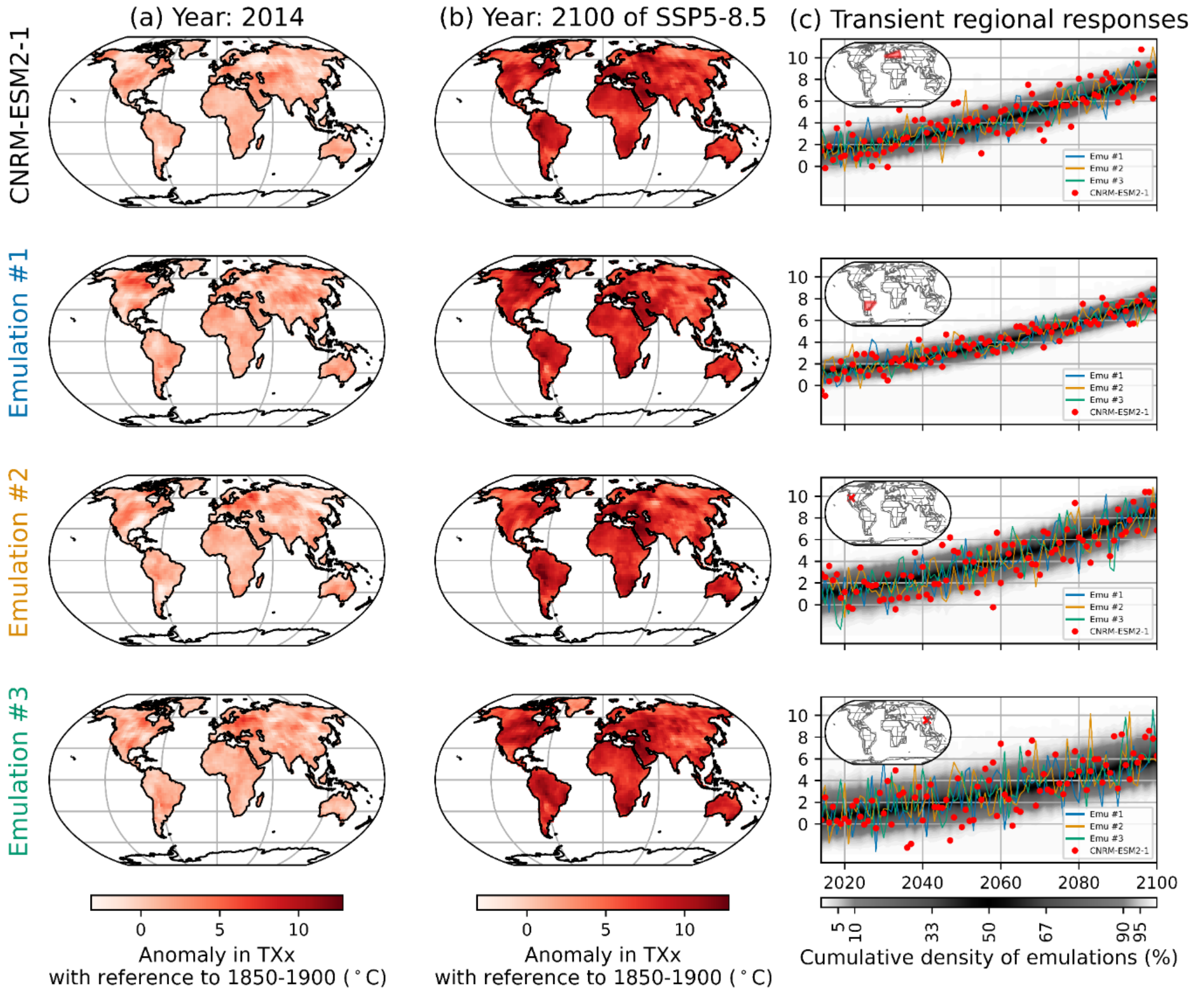

Figure S.33. Same as Figure S.8, but with CNRM-ESM2-1. Note that the scales are adapted to this ESM.

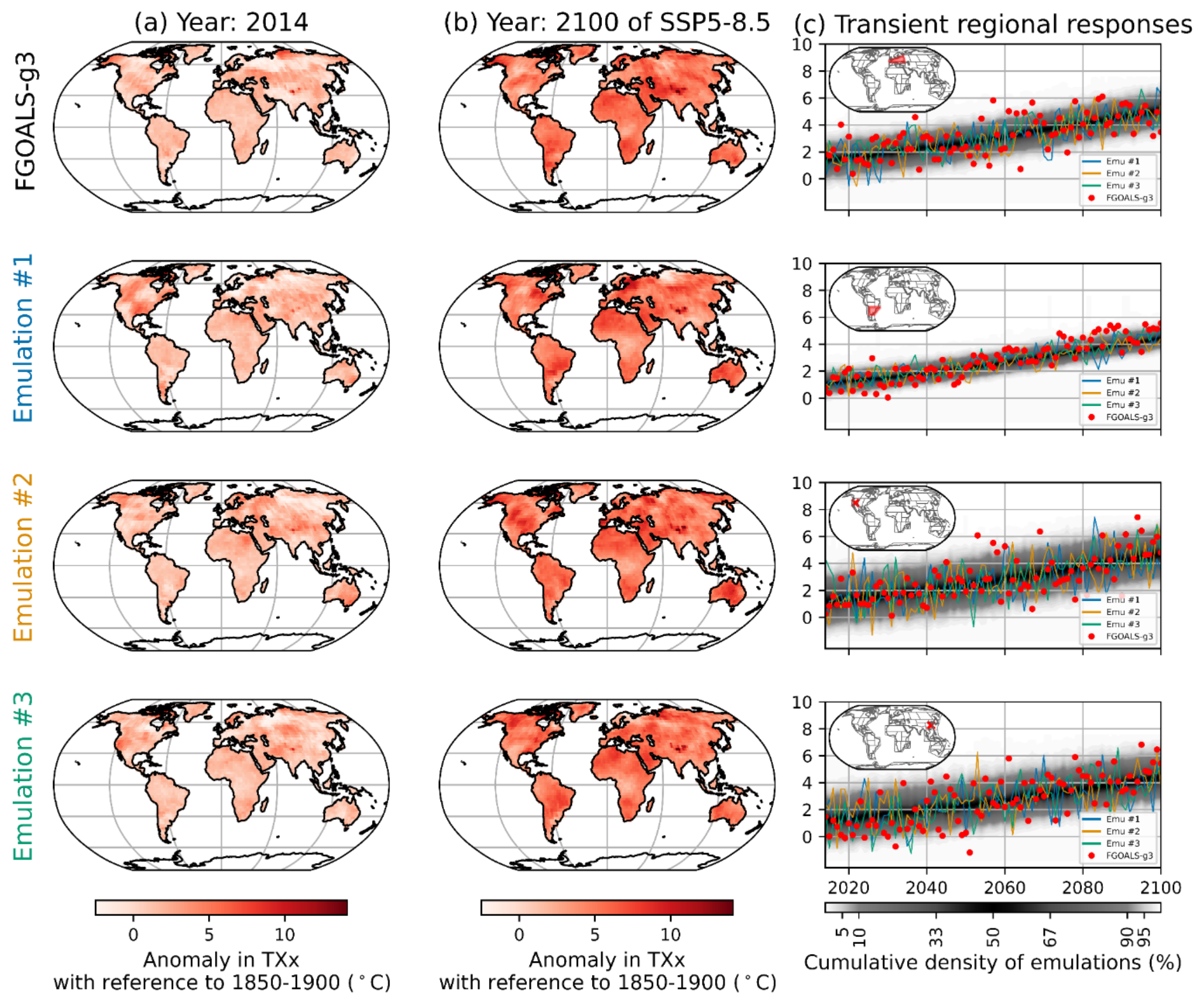

Figure S.34. Same as Figure S.8, but with FGOALS-g3. Note that the scales are adapted to this ESM.

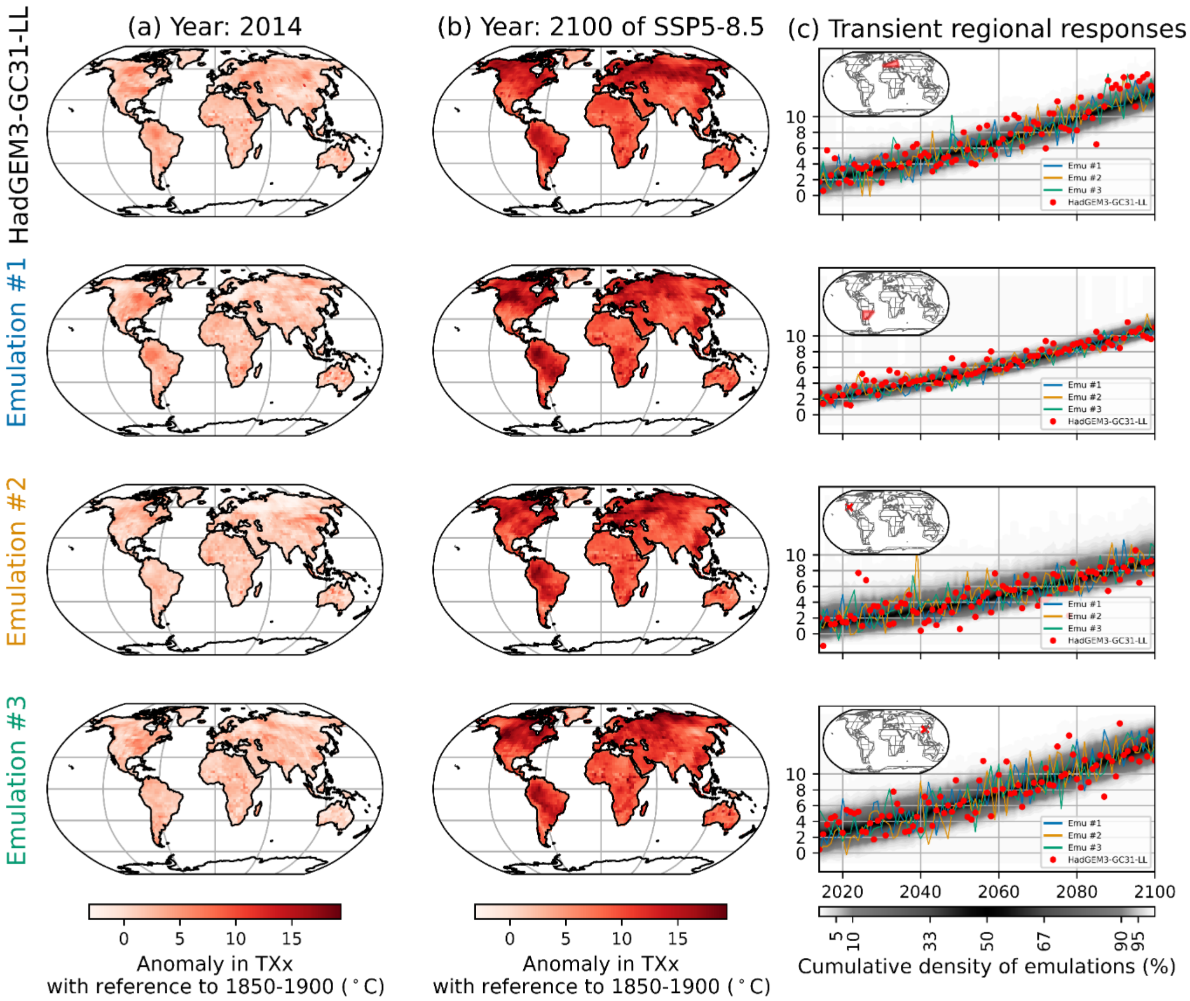

Figure S.35. Same as Figure S.8, but with HadGEM3-GC31-LL. Note that the scales are adapted to this ESM.

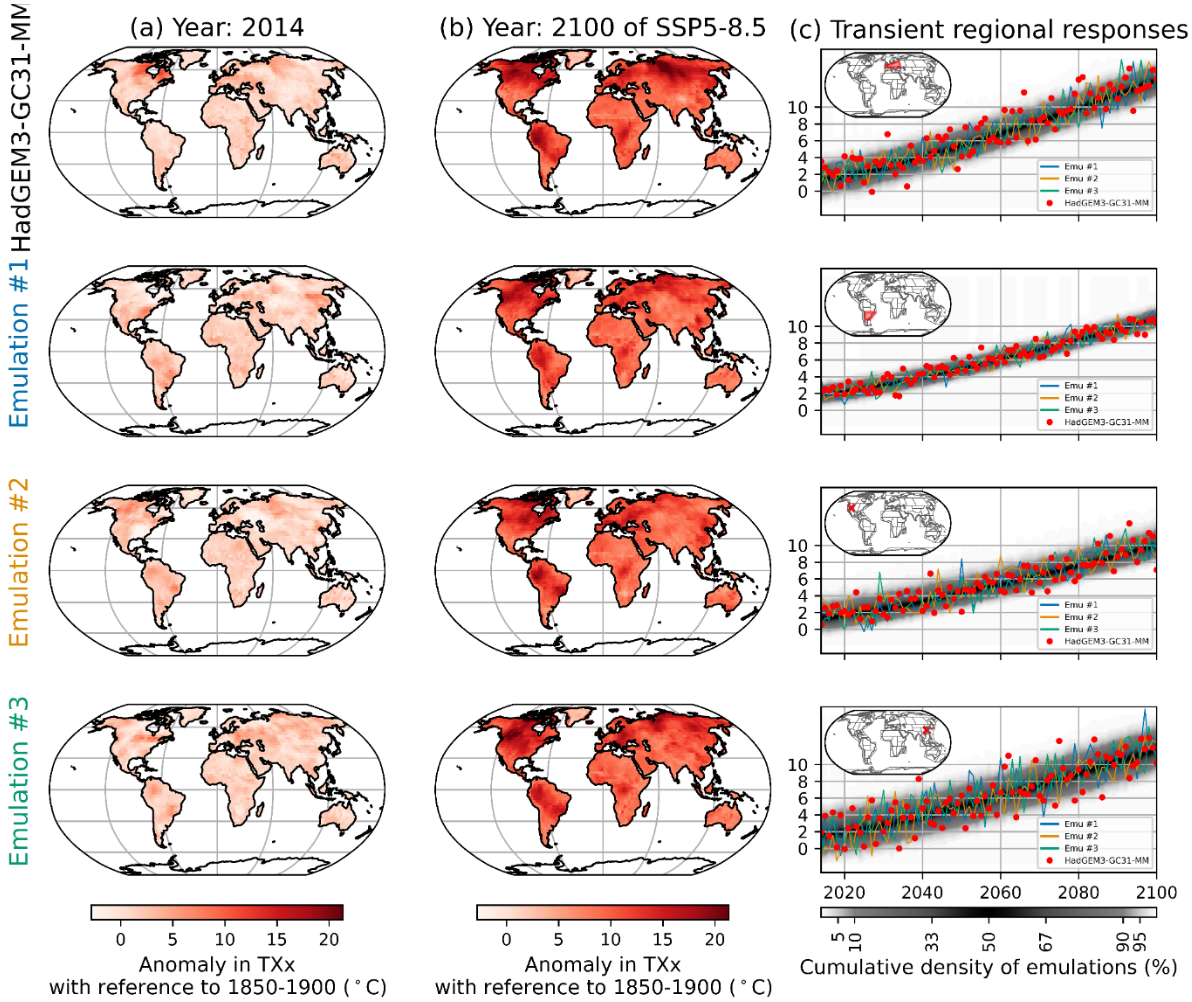

Figure S.36. Same as Figure S.8, but with HadGEM3-GC31-MM. Note that the scales are adapted to this ESM.

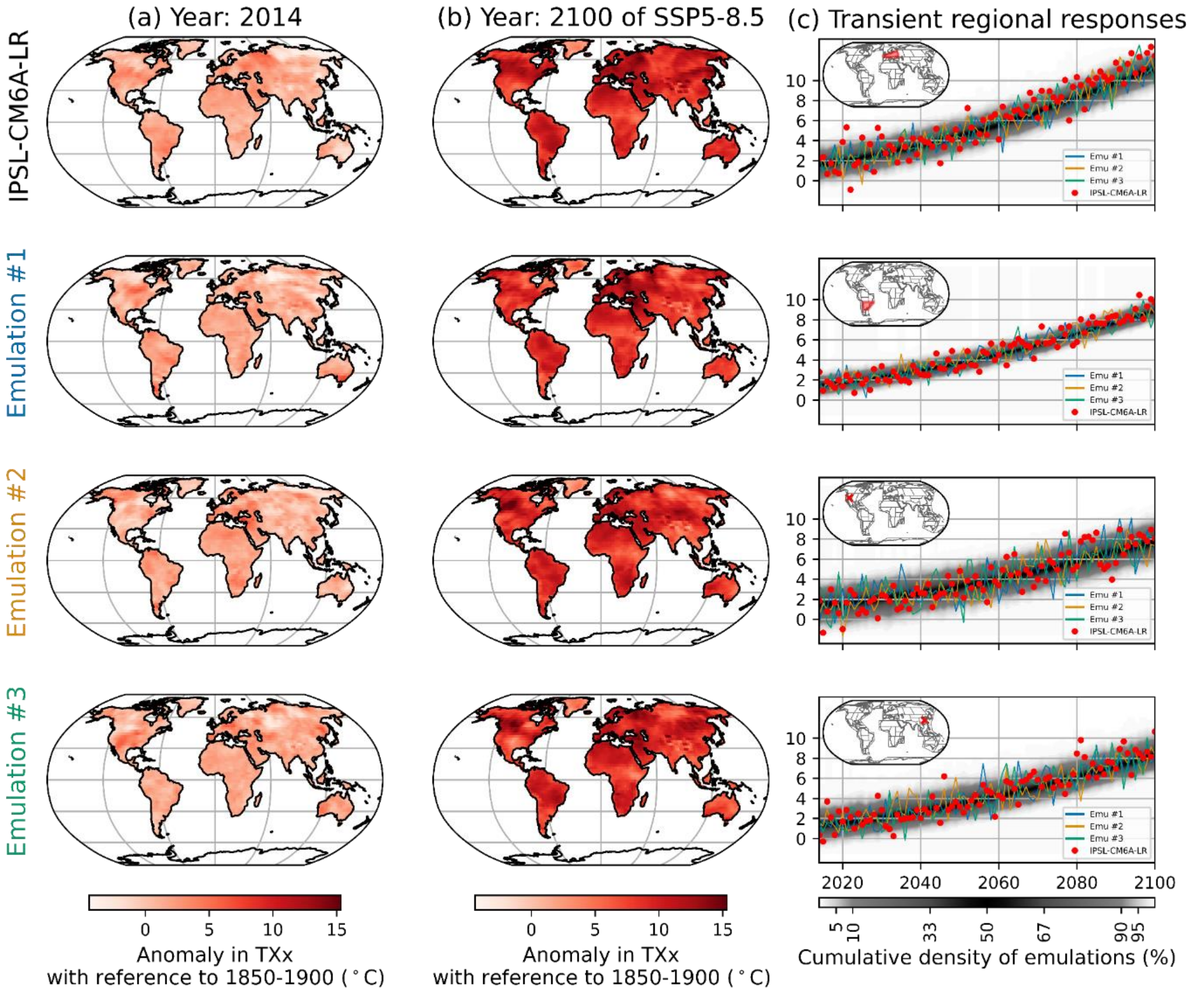

Figure S.37. Same as Figure S.8, but with IPSL-CM6A-LR. Note that the scales are adapted to this ESM.

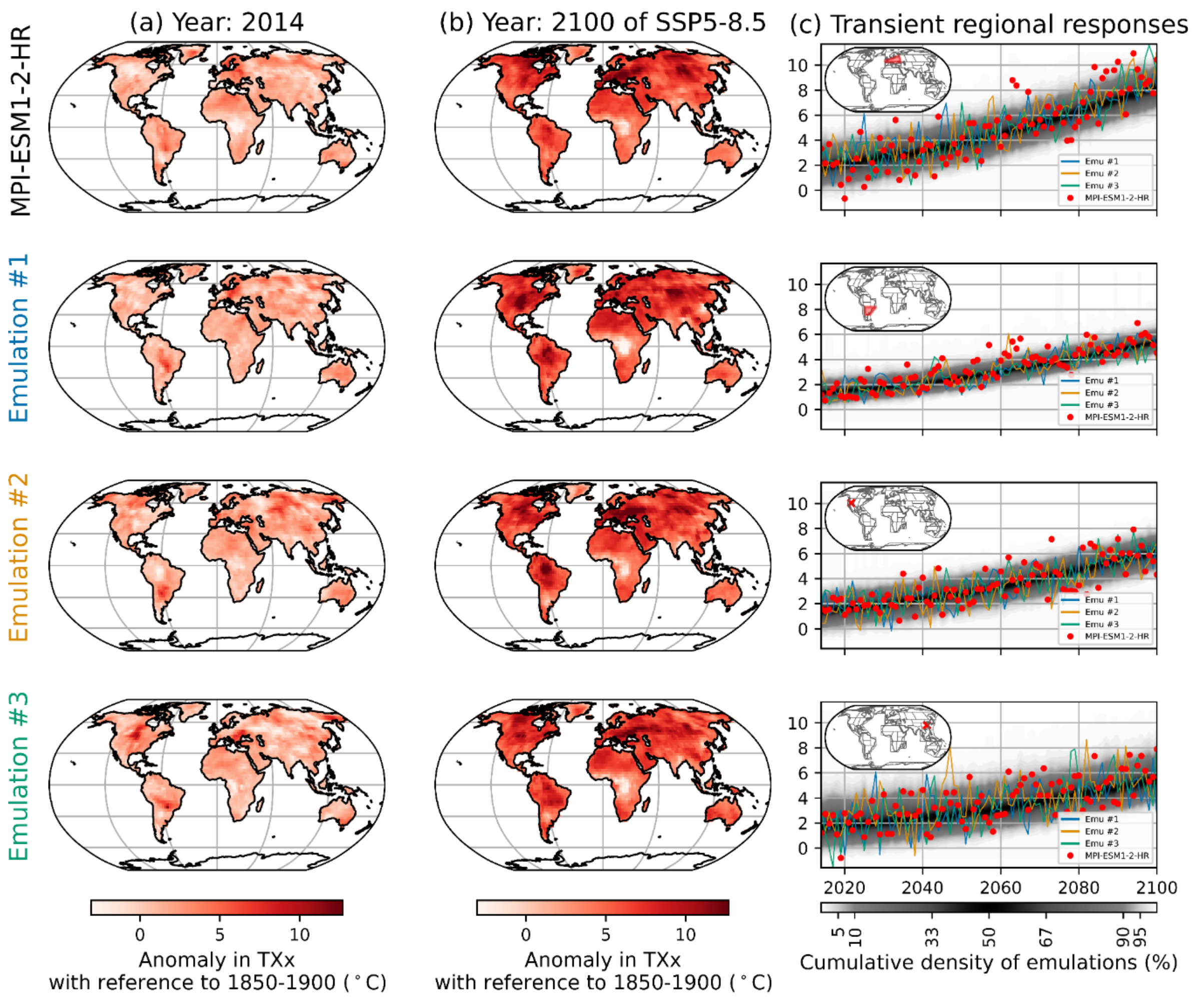

Figure S.38. Same as Figure S.8, but with MPI-ESM1-2-HR. Note that the scales are adapted to this ESM.

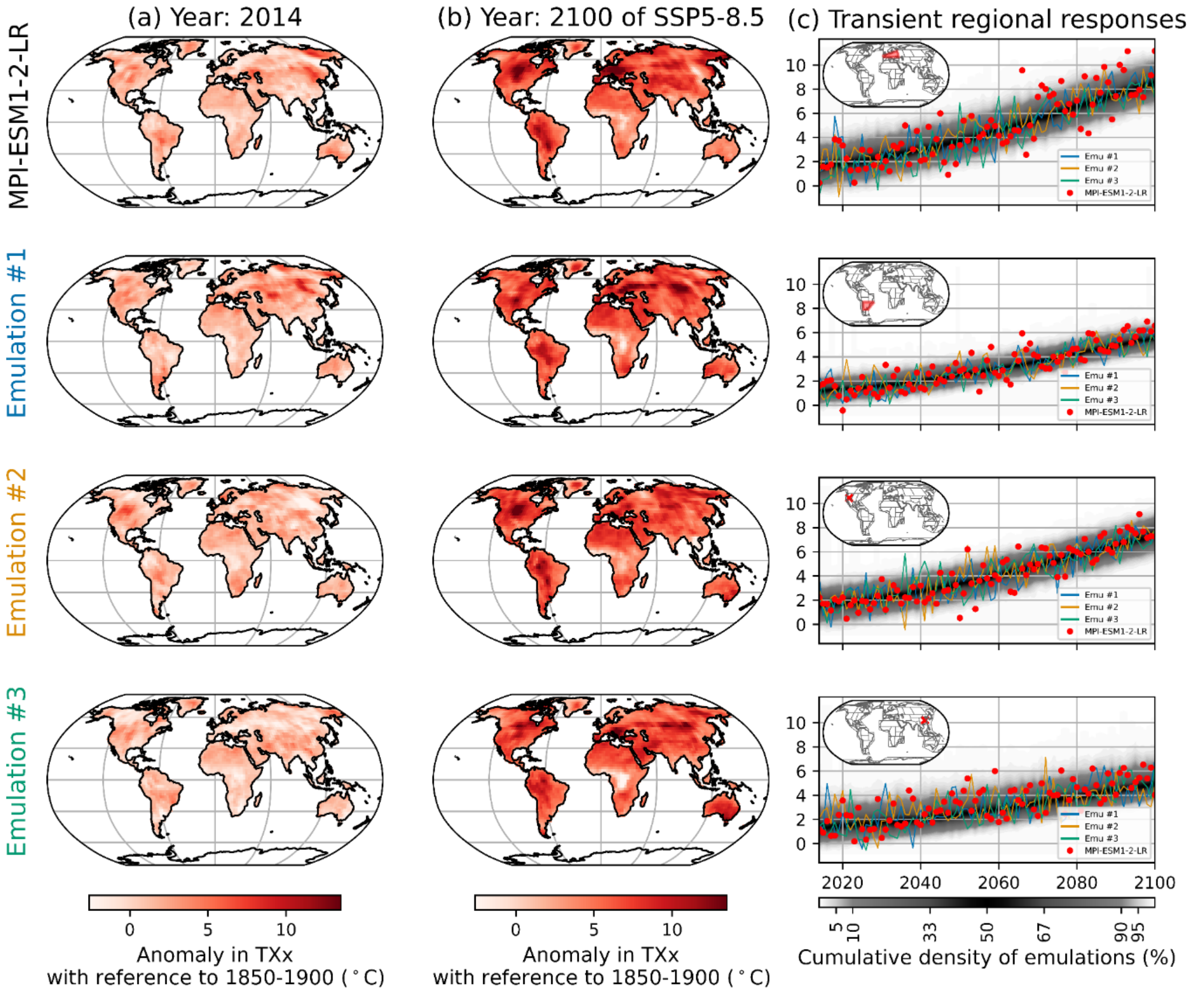

Figure S.39. Same as Figure S.8, but with MPI-ESM1-2-LR. Note that the scales are adapted to this ESM.

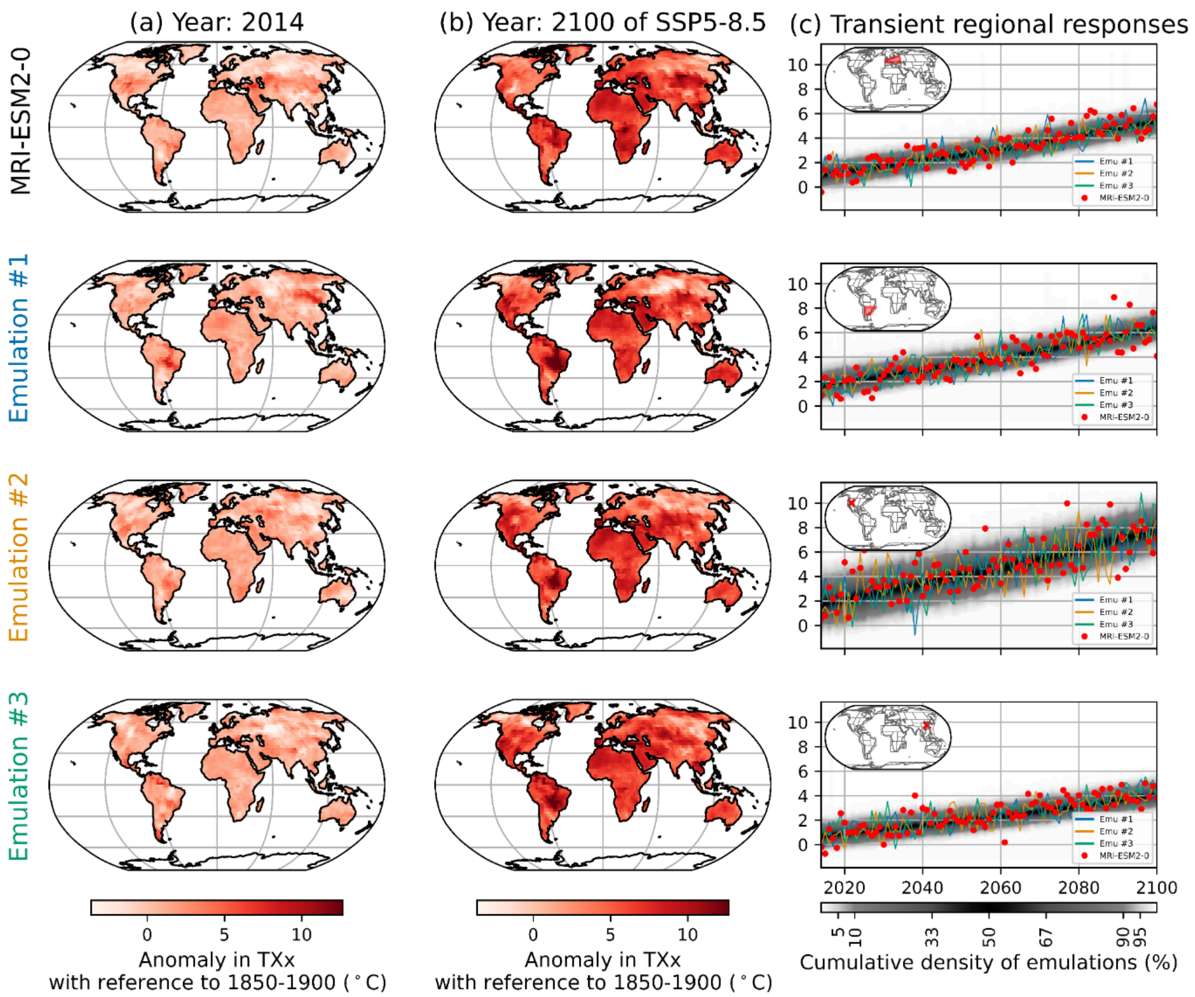

Figure S.40. Same as Figure S.8, but with MRI-ESM2-0. Note that the scales are adapted to this ESM.

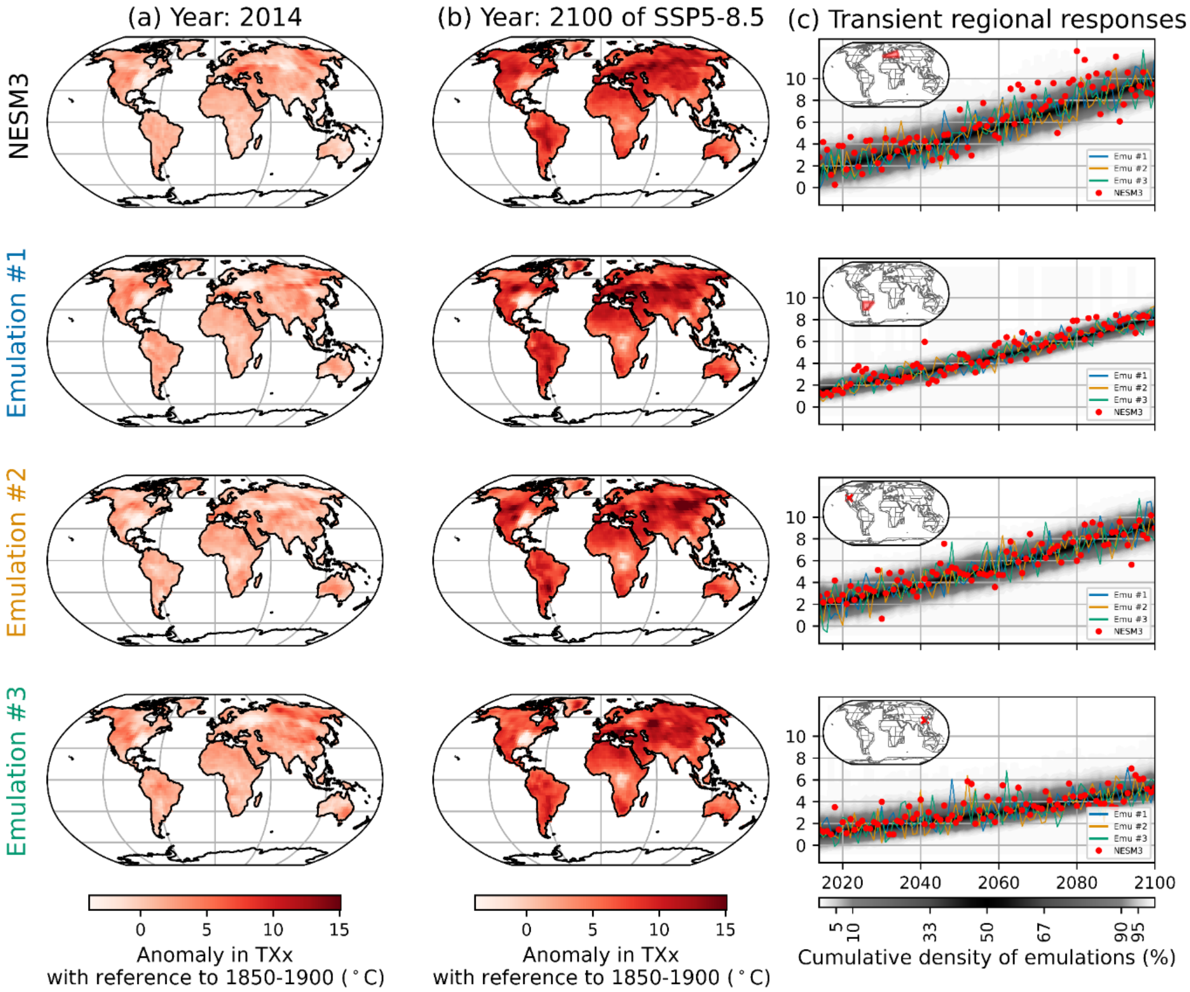

Figure S.41. Same as Figure S.8, but with NESM3. Note that the scales are adapted to this ESM.

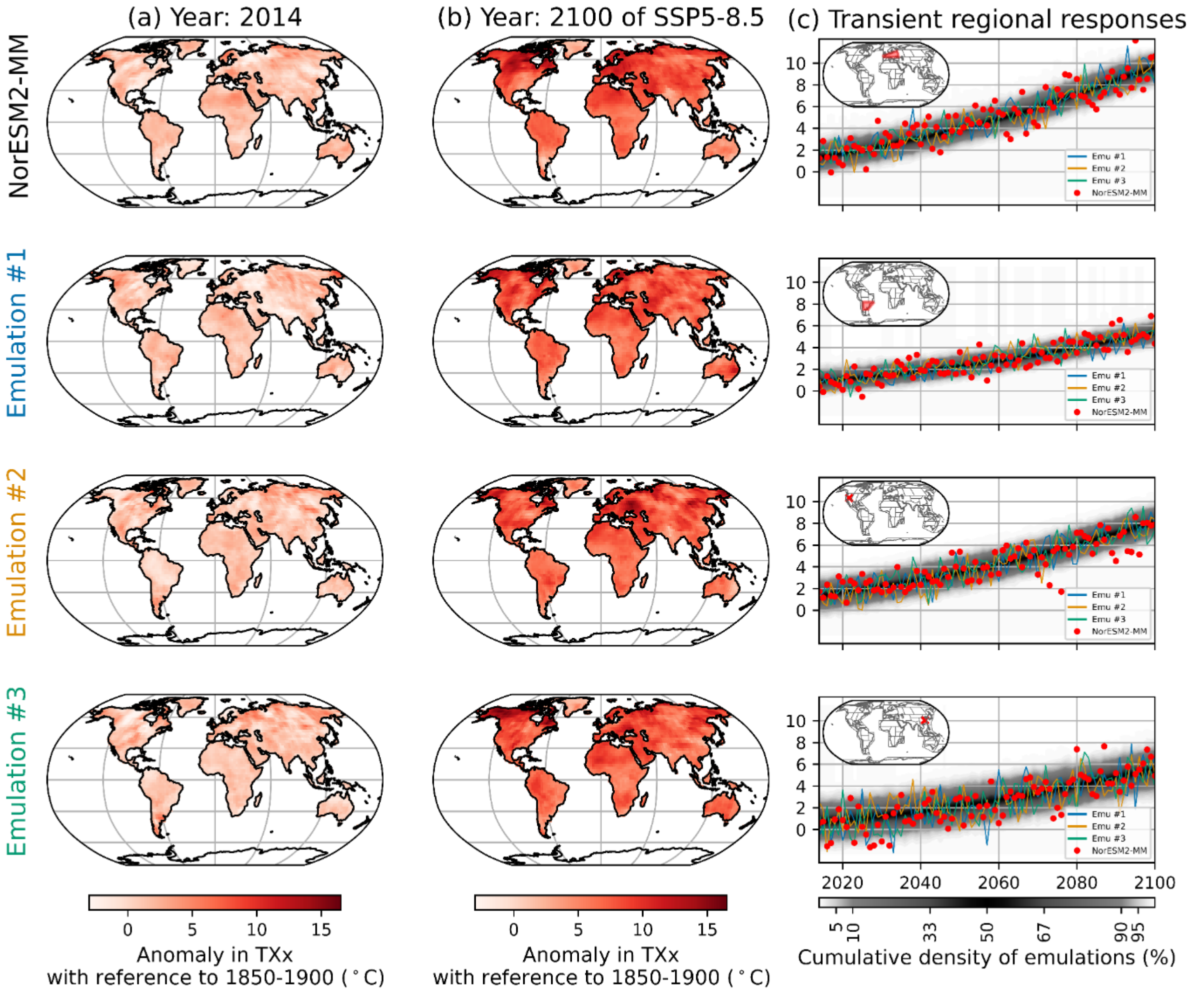

Figure S.42. Same as Figure S.8, but with NorESM2-MM. Note that the scales are adapted to this ESM.

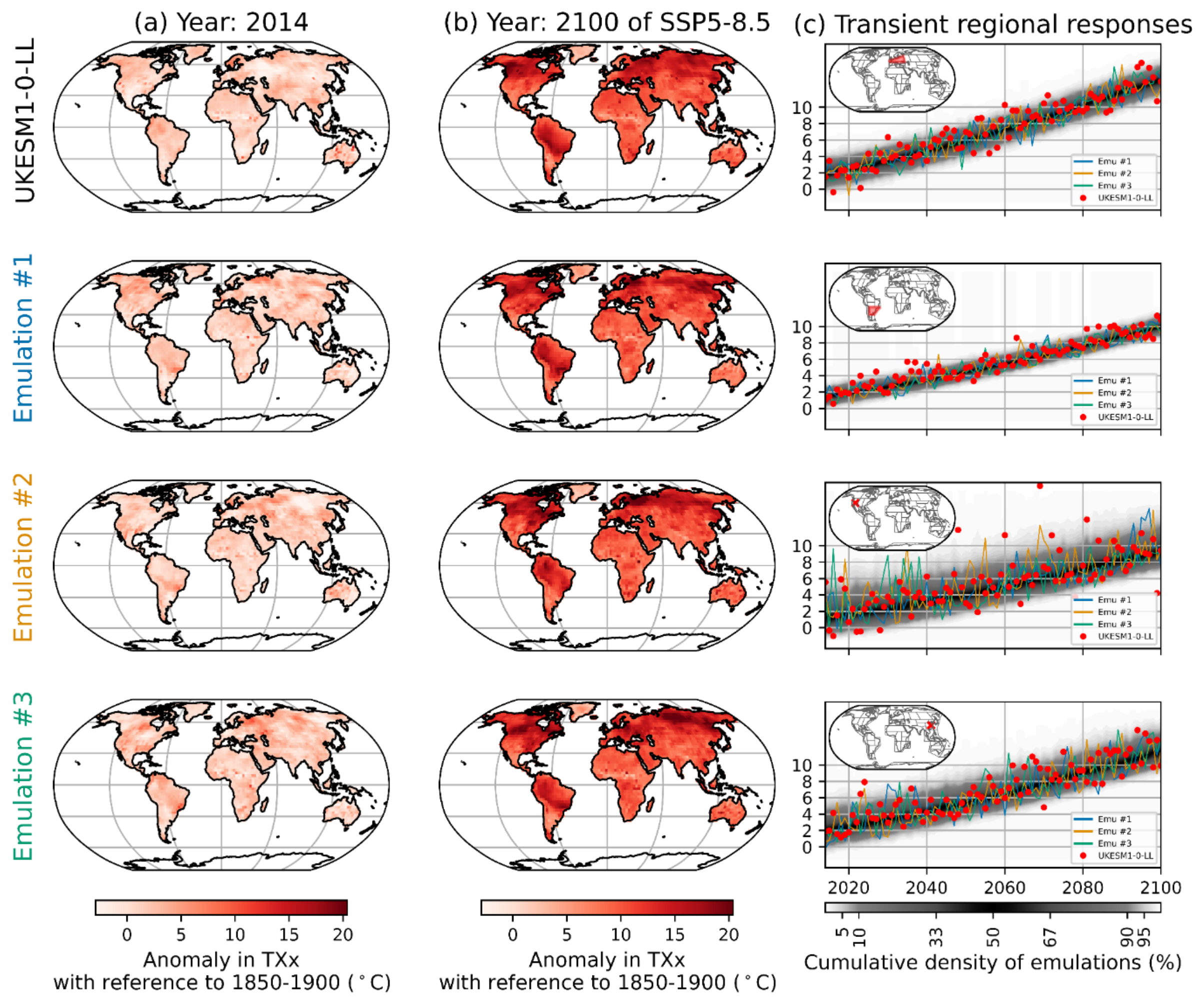

Figure S.43. Same as Figure S.8, but with UKESM1-0-LL. Note that the scales are adapted to this ESM.

ESM	Simulations available	Ensemble member used
ACCESS-CM2	*historical, ssp126, ssp245, ssp370, ssp585*	r1i1p1f1
ACCESS-ESM1-5	*historical, ssp126, ssp245, ssp370, ssp585*	r1i1p1f1
AWI-CM-1-1-MR	*historical, ssp126, ssp245, ssp370, ssp585*	r1i1p1f1
CanESM5	*historical, ssp119, ssp126, ssp245, ssp370, ssp585*	r1i1p1f1
CMCC-CM2-SR5	*historical, ssp126, ssp245, ssp370, ssp585*	r1i1p1f1
CNRM-CM6-1	*historical, ssp126, ssp245, ssp370, ssp585*	r1i1p1f2
CNRM-CM6-1-HR	*historical, ssp126, ssp585*	r1i1p1f2
CNRM-ESM2-1	*historical, ssp119, ssp126, ssp245, ssp370, ssp585*	r1i1p1f2
FGOALS-g3	*historical, ssp119, ssp126, ssp245, ssp370, ssp585*	r1i1p1f1
HadGEM3-GC31-LL	*historical, ssp126, ssp245, ssp585*	r1i1p1f3
HadGEM3-GC31-MM	*historical, ssp126, ssp585*	r1i1p1f1
IPSL-CM6A-LR	*historical, ssp119, ssp126, ssp245, ssp370, ssp585*	r1i1p1f1
MPI-ESM1-2-HR	*historical, ssp126, ssp245, ssp370, ssp585*	r1i1p1f1
MPI-ESM1-2-LR	*historical, ssp126, ssp245, ssp370, ssp585*	r1i1p1f1
MRI-ESM2-0	*historical, ssp119, ssp126, ssp245, ssp370, ssp585*	r1i1p1f1
NESM3	*historical, ssp126, ssp245, ssp585*	r1i1p1f1
NorESM2-MM	*historical, ssp126, ssp245, ssp370, ssp585*	r1i1p1f1
UKESM1-0-LL	*historical, ssp119, ssp126, ssp245, ssp370, ssp585*	r1i1p1f2

Table S.1: ESMs selected for emulation, based on the availability of data.
